# A facile synthesis of cysteine-based diketopiperazine from thiol-protected precursor

**DOI:** 10.1098/rsos.180272

**Published:** 2018-06-20

**Authors:** Di Zhang, Wayne Wang

**Affiliations:** 1 Department of Chemistry, Shanxi Medical University, 56 Xinjian South Road, Taiyuan, Shanxi 030001, China; 2 Department of Chemistry, Carleton University, 1125 Colonel by Drive, Ottawa, Ontario, Canada K1S 5B6

**Keywords:** l-cysteine, diketopiperazines, diastereomer, chiroptical

## Abstract

l-Cysteine is one of the most promising biomass-based building blocks with great potential applications. Herein, we report a versatile synthetic route to produce cysteine-based 2,5-diketopiperazine (DKP) with good yield from the thiol-ene click reaction of l-cysteine and commercially available acrylates, followed by dimerization of the amino acid intermediates. The achieved DKP diastereomers were successfully separated and fully characterized by spectroscopic methods. Moreover, the chiroptical property of DKP in the presence of various metal ions was investigated by circular dichroism spectroscopy. The potential application of the optically active cysteine-based DKP as a chiral probe for detection of silver ion in water has been demonstrated.

## 1. Introduction

The use of biomass-based feedstock to replace fossil fuel-based chemicals is a current main tendency of research and commercial activities [1]. l-Cysteine with abundant resource, is one of the renewable and desirable alternatives. It is readily available on a commercial scale and conventionally derived from hydrolysis of poultry feathers or human hair. It is also available now by large-scale microbial fermentation [2]. As a unique amino acid, l-cysteine possessing a highly reactive thiol moiety, not only has the property and reactivity of the basic amino acid, but also can undergo the famous thiol-ene click reaction under a mild condition with high efficiency and orthogonality [3]. Moreover, thiol and amino acid groups readily form the chelating complexes with silver, lead, copper and other metal ions [4]. l-Cysteine is therefore a versatile naturally-occurring material with a great potential for use as a building block in the synthesis of multifunctional compounds.

2,5-Diketopiperazines (DKPs) are derived directly from amino acids and are also a class of naturally occurring compounds existing in a variety of foods and organisms [5]. They are structurally more rigid than linear amino acids, which can lead to the DKP-containing materials with much improved properties [6]. Moreover, owing to their biodegradability and potential biocompatibility, DKPs have been successfully employed in the preparation of green polymers and in the pharmacological industry for drug delivery and other medical purposes [5–8]. Accordingly, it is highly desirable to develop multifunctional compounds derived from cysteine-based DKP. Most DKPs are formed directly through self-condensation of amino acids or esters by heating [9]. However, the over-reactivity of the thiol group in cysteine makes its DKP synthesis time-consuming and low yield, and limited the research on cysteine-based DKP [10]. Thus, the development of cysteine-based DKP is urgent and of great significance.

Herein, we report the synthesis and chiroptical property of cysteine-based DKPs. Since survival of the thiol group is difficult during the DKP formation, we envision a reverse sequence, in which the thiol group in cysteine is protected first. Thus, the synthetic route involves a simple two-step process; a thiol-ene click reaction with commercially available acrylates and subsequent dimerization of the thiol-protected precursor. The cysteine-based DKPs are optically active and its chiroptical properties are affected specifically by silver ions.

## 2. Results and discussion

### 2.1. Synthesis and characterizations of cysteine-based 2,5-diketopiperazines

**Scheme 1.**
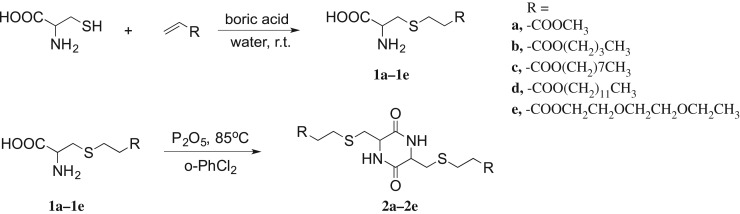
Synthesis of cysteine-based DKPs **2a–2e**.

To avoid the interference of the thiol group during the dimerization process, a thiol-ene click reaction, which is famous for its high efficiency and mild conditions, was first applied to modify the property. A thiol-acrylate Michael addition reaction was reported to proceed in water with almost 100% yield [11]. Thus, the reaction of l-cysteine with acrylates was selected to be carried out in water at ambient temperature for 3 h until a large amount of white solids precipitated out. The *S*-alkylated cysteine derivatives **1a–1e** were then obtained with nearly quantitative yield (scheme 1).

The structures of compounds **1a–1e** were fully characterized by spectroscopic means (electronic supplementary material) [12–13]. Take compound **1a** as an example, in the ^1^H nuclear magnetic resonance (NMR) spectra, the α-proton of amino acid was shifted to the lower field at 3.85 ppm in comparison with l-cysteine. The peaks for the two carbonyl's carbons appear at 175.06 ppm and 172.71 ppm in the ^13^C NMR spectra, further confirming the successful synthesis of s-alkylated cysteine derivative **1a**. In addition, molecular ion peaks in mass spectra (MS) is consistent with its structure. Besides, compounds **1a–1e** are chiral and optically active, as characterized by circular dichroism (CD) spectroscopy. The CD spectra show a peak at 225 nm with a positive Cotton effect (electronic supplementary material).

**Scheme 2.**
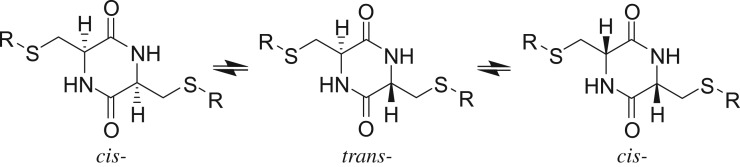
Isomerization of *cis*- and *trans*-DKP isomers.

The synthesis of DKP **2a–2e** involves the dimerization of compound **1a–1e** through intermolecular amide formation and intramolecular lactamization (scheme 2). Countless experiments were carried out to identify the feasible reaction condition including reaction catalysts, solvents and temperatures. First, a series of known coupling reagents for amide bond formation were tested in our work, such as dicyclohexylcarbodiimide, *N*,H'-diisopropylcarbodiimide, and O-benzotriazole-*N,N,N',N'*-tetramethyl-uronium-hexafluorophosphate [14]. However, these peptide catalysts lack activity for intermolecular cyclization and only afforded linear dipeptide. By contrast, P_2_O_5_ has been reported as an effective coupling reagent in several DKP formations and could not only speed up the reaction rate, but also improve the yields significantly [15,16]. Therefore, P_2_O_5_ is an ideal catalyst with features including cost-effective, environmental-friendly and can easily be removed by water washing was used in our work. In terms of the reaction solvent, several factors were taken into account including the solvent boiling points, polarity and inherent natures [17]. Most of the common solvents available in the laboratory were carefully examined. Finally, 1,2-dichlorobenzene which is a polar, protophilic solvent with high-boiling point, was proven to be the only applicable solvent for the cyclization reaction. Accordingly, the dimerization reaction was finally carried out in 1,2-dichlorobenzene in the presence of P_2_O_5_ as a catalyst and dehydrating reagent. The reaction was first test run at different temperatures from 40 to 120°C. It was found that the reaction did not go to completion when the temperature was lower than 80°C and at high temperatures the starting materials and final products tend to decompose. Thus, the reaction was best carried out at 80–110°C.

### 2.2. Stereochemistry of cysteine-based 2,5-diketopiperazine

**Figure 1.**
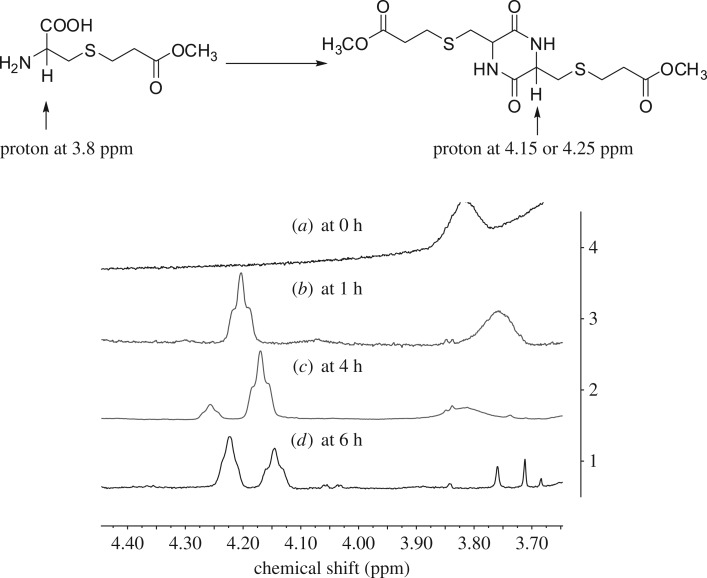
^1^H NMR spectra (300 MHz, DMSO-d_6_) showing the progress of dimerization reaction of compound **1a** to form DKP **2a** at different reaction times.

The course of the dimerization reaction could be monitored by the changes of the α-proton from amino acid in compound **1** at 3.8 ppm in ^1^H NMR spectra. Take compound **1a** as an example, after 1 h of dimerization, a new peak appeared at 4.15 ppm along with the peak at 3.8 ppm (spectrum (*b*) in figure 1), indicating a low degree of conversion at the early stage of reaction (around 1 h). The peak at 4.25 ppm gradually increases as the reaction proceeds (spectrum (*c*) in figure 1). After 6 h, the ^1^H NMR spectrum shows the presence of two intense peaks at 4.25 and 4.15 ppm and absence of the α-proton resonance at 3.8 ppm (spectrum (*d*) in figure 1).

It is known that the dimerization of amino acid leads to the formation and isomerization of the *cis*/*trans*-DKP isomers, where the *cis*-DKP is C_2_-symmetric and the *trans*-isomer is centre-symmetric (scheme 2) [9]. It is assumed that the formation of diastereomers results from the epimerization of the chiral α-proton at high temperature. Thus, at the beginning of the reaction, the formation of ll-isomer predominated because of the presence of a large amount of l-stereo isomeric precursor. As the dimerization reaction proceeds, isomerization between the *cis*-isomer and the *trans*-isomer also takes place, leading to the formation of two diastereomers or ll-/dd- and ld-DKP. Therefore, the peak at 4.25 ppm can be assigned to *trans*-DKP **2a** and the one at 4.15 ppm is from the *cis*-isomer (figure 1). To confirm our hypothesis, research on synthetic DKP from various amino acids were investigated and it is found that little racemization took place in the beginning of the dimerization where only *cis*-isomer presented, and as time went by, the ratio of *cis*/*trans* gradually decreased [9,18]. Moreover, the α-proton of *trans*-DKP is normally located at the lower field region than that of *cis*-DKP in the ^1^H NMR spectra in different literature, which is same as the results of cysteine DKPs and the distinction was attributed to the conformational difference of the cyclic and intramolecular hydrogen bonding [19–21]. Therefore, the assignments of peak at 4.25 ppm to *trans*-DKP **2a** and the one at 4.15 ppm to *cis*-isomer are reasonable.

It was reported that at the higher temperatures the dimerization is faster and more racemization takes place [18]. The same observations were taken in the preparations of cysteine-based DKPs. It was found that there were three possible factors that can influence the diastereomeric ratios, namely temperature, time and catalyst. Therefore, for a given reaction time of 6 h, the effect of P_2_O_5_ catalyst and reaction temperature on the compositions of *cis*/*trans* isomers were investigated for the dimerization of compound **1a**. The ratios of *cis* and *trans* isomers were determined by the ^1^H NMR method and listed in table 1. It was clear that the temperature and catalyst concentration had a significant impact on the reaction outcomes. When there was an increase in the temperature or catalyst concentration, the *trans*-isomer was formed more than the *cis*-isomer or the ld-diastereomeric DKP was a major product.

**Table 1.** Influence of temperature and P_2_O_5_ catalyst on the formation of *cis*/*trans* isomers of DKP **2a.**

run	time (h)	catalyst (%)	temperature (°C)	*cis* : *trans* ratio
1	6	10	80	1 : 0.8
2	6	20	80	1 : 1.2
3	6	10	120	1 : 1.5

It is known that diastereomers have different physical and chemical properties, and thus are worthy of separation. Chromatography is the most popular method of separating DKP diastereomers [22,23]. Because the DKPs from **2b** to **2e** are more soluble in organic solvents with the increase of the chain lengths, these compounds could be separated by column chromatography. But interestingly, the diastereomeric isomer of DKP **2a** was able to be separated by selective precipitation in methanol. Owing to the different conformations of the rigid DKP rings and hydrogen bond interactions, the dominant isomer was prone to aggregate in methanol and precipitate afterwards. Thus, the desired pure isomer of DKP **2** could be easily obtained through controlling the ratio of the diastereomers formed during the dimerization process, followed by a simple filtration work-up. For example, when the reaction stopped after 1 h, the *cis*-isomer of DKP **2a** was the main product. Since it could self-aggregate and precipitate from methanol, the pure *cis*-isomer was successfully isolated by simple filtration. Likewise, when the *trans*-isomer became the major component, the product collected from methanol precipitation would be pure *trans*-isomer. Moreover, it was found that the isomers of compound **2a** had obvious differences in physical properties. For example, the *cis*-isomer has a melting point at 220°C, which is slightly higher than its *trans*-counterpart. In addition, the solubility of *trans* **2a** in dichloromethane is better than its *cis*-counterpart, which was attributed to the fact that the *cis*-isomer has greater tendency in self-assembling through intermolecular hydrogen bonds (N−H^…^O) between adjacent molecules.

**Figure 2.**
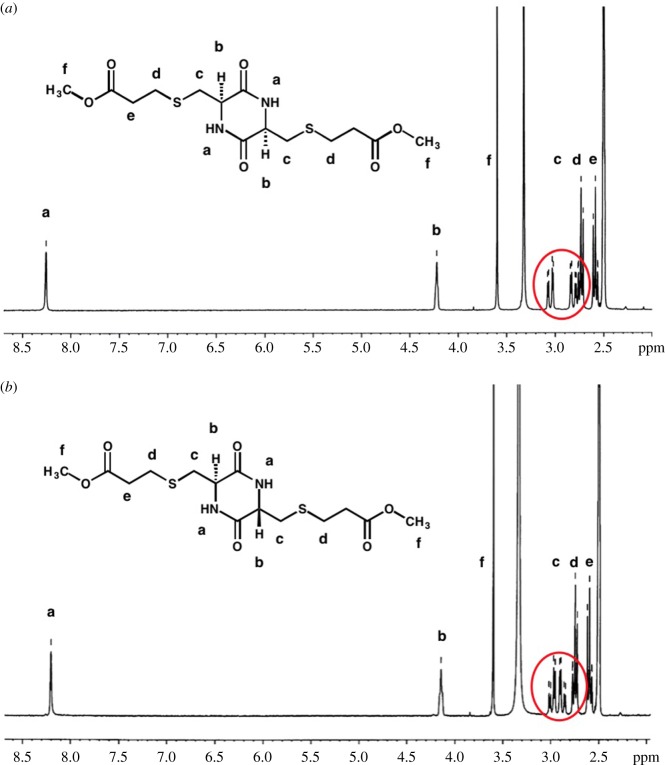
^1^H NMR (300 HMz, dimethylsulfoxide-d_6_) spectra of (*a*) *cis*-**2a** and (*b*) *trans*-**2a**.

The diastereomer structures of DKP **2** were fully characterized by spectroscopic means (electronic supplementary material). Take the ^1^H of DKP **2a** as an example (figure 2). A clear difference was observed from the signals of CH_2_ in amino acid, where the peaks of the *cis*-isomer was more narrow, which was attributed to the conformational differences of the cyclic rings and intramolecular hydrogen bonds. Therefore, to distinguish the diastereomers of cysteine-based DKP, one could simply compare the specific data in ^1^H NMR spectra.

Studies on DKPs having aliphatic side chains revealed that the information on isomer structures could be obtained from the ^3^J coupling constants of H-^α^C-^β^C-H. In NMR analysis, *cis*-DKP normally has a larger coupling constant compared to the *trans*-isomer [24]. Thus, the ^3^J coupling constants of H-^α^C-^β^C-H are 4.8 or 4.2 Hz for the *cis*-isomer and 4.2 or 3.3 Hz for the *trans*-isomer of DKP **2a**, is also consistent with previous analysis. Moreover, the results from heteronuclear single quantum coherence, correlation spectroscopy NMR experiments draw the same conclusion on the assignment of *cis*- and *trans*-isomers. It has been reported that the DKP ring could exist in a flat or a slightly puckered boat conformation. Furthermore, the *cis*-isomer normally exists in twist-boat conformation, while the *trans*-isomer prefers a planar ring [25–27]. Since DKP conformations can aid the structure assignments, nuclear overhauser effect spectroscopy and nuclear overhauser effect (NOE) measurements were applied to identify the conformations. It can be seen that a 16.5% NOE on NH was observed for the *cis*-isomer and 9.1% NOE was registered for the *trans*-isomer (electronic supplementary material). The observations are accordance with the speculation of DKP conformations and further confirms the stereochemistry distribution.

### 2.3. Chiroptical property of cysteine-based 2,5-diketopiperazine

**Figure 3.**
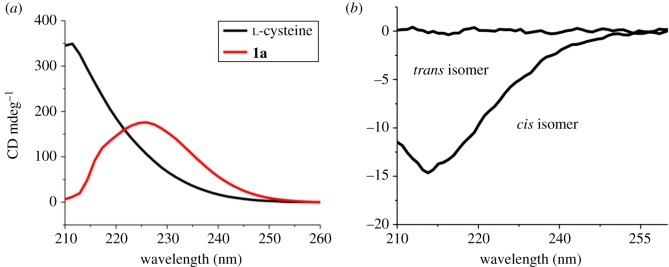
CD spectra of (*a*) l-cysteine and compound 1a in water; (*b*) *cis*-**2a** and *trans*-**2a** in water.

l-Cysteine contains a chiral carbon and is capable of producing chiral derivatives. Many l-cysteine derivatives are known to have chiroptical activity for metal sensing application [28,29]. Therefore, the chiroptical property of the DKP-containing compound **2a** was measured by CD spectroscopy. First, it was found that compound **1a** is chiral and optically active, as characterized by CD spectroscopy (figure 3*a*). The CD spectra of compound **1a** shows a peak at 225 nm with a positive Cotton effect. This result is consistent with the point that there is no racemization detected during the synthesis of *S*-alkylated compound [30]. Then, CD measurements on DKP diastereomers were performed. It is known that the C_2_-symmetric *cis*-DKP is optically active and centre-symmetric *trans* isomer is not optically active (scheme 2). When the dimerization reaction of compound **1a** was terminated at the early stage where the *cis*-isomer was mainly produced, the isolated *cis* isomer of DKP **2a** was optically active with a specific optical rotation of −88° (C = 0.05, CH_3_OH). Its CD spectrum displays a negative Cotton effect at 220 nm (figure 3*b*), which can be assigned to the n-π* transition of the amide group with reference to other known cyclic amino acids [22]. As expected, the *trans* isomer of DKP **2a** shows no optical activity (figure 3*b*). However, when the dimerization reaction went to nearly completion, the final products became totally optical inactive, indicating the complete racemization of each chiral isomer.

**Figure 4.**
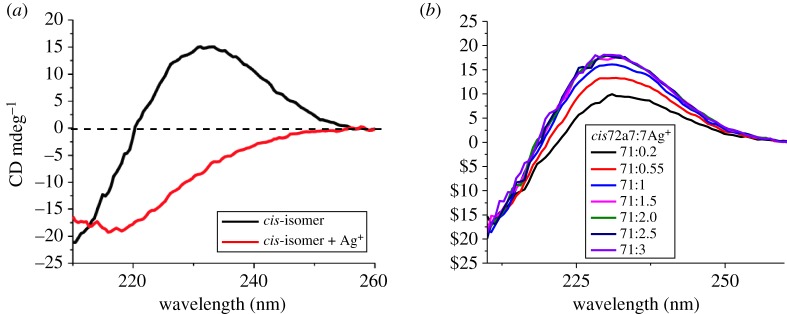
CD spectra of (*a*) *cis*-**2a** with Ag^+^; (*b*) *cis*-**2a** with various molar ratios of Ag^+^.

Cysteine-based DKP contain the amine, carbonyl and sulfide groups that are able to bind some metal ions [31]. If the metal binding is selective or specific and leads to a significant change in chiroptical property, the optically active cysteine-based DKP is potentially useful for chiroptical sensor of certain metal ions. The binding behaviour of the isolated optically active *cis* isomer of **2a** with different metal ions was studied by CD measurement. Solutions of various metal ions (Zn^2+^, Cu^+^, Pd^2+^, Ni^2+^, Mg^2+^, Ca^2+^, Ba^2+^, Co^2+^, Li^+^, Mn^2+^, Pb^2+^, Na^+^ and Ag^+^) were added and CD spectra were taken immediately afterwards. It was found that for the un-chelated ions, for example Pt^2+^, there was no signal changing observed. For most of the chelated ions, chelation induced only slightly shift or decrease in CD signals, for example, Cu^2+^. When Cu^2+^ was added, the CD band shifted from 217 nm to 233.7 nm accompanying a significant decrease (electronic supplementary material). But the chelation with silver ions induced a significant change in CD spectra (figure 4*a*). As shown, upon addition of silver ions (1 × 10^−5^ M), a new CD band with an opposite sign appeared at 230 nm. In addition, by changing the molar ratios of Ag^+^ to the optically active *cis*-isomer of **2a**, the maximal binding ratio of 1 : 2 was found (figure 4*b*).

Sensing and monitoring silver ions becomes important as silver pollution in the environment increasingly threatens a broad range of microorganisms and marine invertebrates. Various chemo-sensors have been developed for Ag^+^ detection in aqua systems which are normally based on organic fluorophores and semiconductor quantum dots. However, these chemo-sensors have some drawbacks such as hard preparative, low water solubility, and/or poor selectivity toward Ag^+^ [32]. Thus, water-soluble, optically active cysteine-based DKP could be used as a sensitive CD probe for detection of silver ions in water with a concentration as low as 1 × 10^−5^ M. In addition, since the cysteine-based DKP compounds are potentially biodegradable, the DKP could also function as an environmental-friendly sensor.

## 3. Conclusion

Facile synthesis of cysteine-based DKP from thiol-protected precursors, mainly s-alkylated cysteine-derivatives, were demonstrated, where a thiol-acrylae Michael addition reaction was used to suppress the over-reactivity of the thiol group. A series of DKP containing small molecules were successfully synthesized with the existence of diastereoisomers. The separations of DKP diastereoisomers were achieved successfully. Moreover, the chiroptical activity of the diastereoisomers combined with the isolated enantiomers of DKP compounds has the potential to be used for sensing silver ions in aqua systems.

## Supplementary Material

Supporting Information

## Data accessibility

We have submitted an electronic supplementary document that includes all the data supporting the results in our article.
