# Supplementary material for: A facile synthesis of cysteine-based diketopiperazine from thiol-protected precursor
Source: R Soc Open Sci. 2018 Jun 20;5(6):180272. doi: 10.1098/rsos.180272 (PMC6030340; doi:10.1098/rsos.180272)

**Supporting Information**

# A facile synthesis of cysteine-based diketopiperazine from thiol-protected precursor

# Di Zhang,*^a^ Wayne Wang*^b^

# ^a^ Department of Chemistry, Shanxi Medical University, 56 Xinjian South Road, Taiyuan, Shanxi, China 030001

# ^b^ Department of Chemistry, Carleton University, 1125 Colonel by Drive, Ottawa, Ontario, Canada K1S 5B6

# E-mail: zhangdi0801@live.cn （Di Zhang）

# wayne_wang@carleton.ca (Wayne Wang)

**Table of Contents**

Experimental section 2

[Figure S1. CD spectra of L-cysteine and 1a 6](#_Toc403657305)

[Figure S2. CD spectra upon addition of different metal ions into cis 2a at 1:1 molar ratio 6](#_Toc403657306)

[Figure S3. CD spectra of cis 2a upon addition of Cu^2^+ at different molar ratio 7](#_Toc403657307)

[Figure S4. CD spectra of cis 2a upon addition of Ag+ at different molar ratio 7](#_Toc403657308)

[Spectra of 1a and 2a 8](#_Toc403657309)

[Spectra of 1b and 2b 16](#_Toc403657310)

[Spectra of 1c and 2c 20](#_Toc403657311)

[Spectra of 1d and 2d 23](#_Toc403657312)

[Spectra of 1e and 2e 27](#_Toc403657313)

## **Experimental section**

### Materials

L-cysteine, methyl acrylate, butyl acrylate, lauryl acrylate, di(ethylene glycol) ethyl ether acrylate, phosphorous pentoxide, 1,2-dichlorobenzene, boric acid, 1-dodecene, 1-undecene were purchased from Sigma Aldrich Chemicals Canada. Octyl acrylate was purchased from MP Biomedicals. The water used in this work was purified using a Millipore™ Milli-Q™ Advantage A10 water purification system. Column chromatography was done using silica gel (Silicycle Chemical Division, 70–230 mesh) as the stationary phase.

### Measurements

^1^H, ^13^C, COSY, NOESY, HSQC, NOE NMR spectra were measured on a Bruker Avance Digital 300 MHz spectrometer at ambient temperature using tetramethylsilane as an internal standard. Mass spectra were performed with a Micromass Quattro LC ESI (EI). Fisher-Johns melting point apparatus was used to test the melting points (mp). CD spectrum was recorded on Olis Circular Dichroism Spectrophotometers and the wavelength starting from 210 nm. Specific rotation was performed on Autopol IV Automatic Polarmeter at 23 ^o^C in methanol.

### Synthesis

**General procedure for cysteine reacting with acrylate:**

Cysteine (1.21 g, 10.0 mmol) and boric acid (0.12 g, 10 wt%) were dissolved in 20 mL of distilled water in 50 mL round-bottom flask. Acrylate was then added under argon atmosphere. The mixture was stirred at ambient temperature for 3 h. After the reaction was completed, the solution was dropped into acetone with vigorous stirring and the product was collected by suction filtration.

**1a:** ^1^H NMR (300 MHz, D_2_O): 3.87-3.83 (dd, *J*=7.5, 4.2 Hz, 1H), 3.63 (s, 3H), 3.09-2.92 (q, *J*=14.7, 7.2, 4.4Hz, 2H), 2.80-2.75 (dd, *J*=8.4, 1.8 Hz, 2H), 2.68-2.63 (dd, *J*=8.4, 2.1 Hz 2H); ^13^C NMR (75 MHz, D_2_O): 175.06, 172.71, 53.50, 52.32, 33.84, 32.07, 26.32; m.p.: 220 ^o^C.

**1b:** ^1^H NMR (300 MHz, D_2_O + HCl): 4.13-4.09 (dd, *J*=7.2, 4.8 Hz, 1H), 3.92-3.88 (t, *J*=6.6 Hz, 2H), 3.04-2.86 (q, *J*=15, 7.2, 4.5 Hz, 2H), 2.66-2.61 (dd, *J*=7.8, 1.2 Hz, 2H), 2.61-2.46 (dd, *J*=7.5, 0.9 Hz, 2H), 1.42-1.33 (m, 2H), 1.17-1.05 (tq, *J*=7.5 Hz, 2H), 0.64 (t, *J*=7.5 Hz, 3H); ^13^C NMR (75 MHz, D_2_O + HCl): 174.53, 170.13, 65.57, 51.97, 34.01, 31.03, 29.68, 26.54, 18.34, 12.76; m.p.: 220 ^o^C.

**1c:** ^1^H NMR (300 MHz, DMSO-d_6_+HCl): 4.10-4.08 (d, *J*=5.1 Hz, 1H), 3.97-3.87 (dd, *J*=5.7, 1.2 Hz, 2H), 3.09-2.91 (d, *J*=6 Hz, 2H), 2.77-2.57 (ddd, *J*=53.4, 13.5, 6.9 Hz, 4H), 1.52-1.46 (m, 2H), 1.32-1.21 (m, 8H), 0.85-0.78 (m, 6H); ^13^C NMR (75 MHz, DMSO-d_6_ + HCl): 171.90, 169.88, 66.58, 52.31, 38.52, 34.56, 31.60, 30.15, 28.72, 27.37, 23.60, 22.81, 14.36, 11.24; M.P.: 190 ^o^C; EI-MS: C_14_H_27_NO_4_S for M, calculated: 305.2, found 305.2.

**1d:** ^1^H NMR (300 MHz, DMSO-d_6_ + HCl): 4.10-4.08 (d, *J*=5.1 Hz, 1H), 3.97-3.87 (dd, *J*=5.7, 1.2 Hz,, 2H), 3.09-2.97 (d, *J*=6 Hz, 2H), 2.78-2.57 (ddd, *J*=53.4, 13.5, 6.9 Hz, 4H), 1.52-1.46 (m, 2H), 1.32-1.24 (m, 18H), 0.83 (m, 3H); ^13^C NMR (75 MHz, DMSO-d_6_ + HCl): 171.85, 169.93, 64.56, 52.32, 34.55, 31.73, 31.69, 29.50, 29.43, 29.40, 29.35, 29.14, 29.07, 28.52, 27.34, 25.77, 22.53, 14.41; M.P.: 185 ^o^C; EI-MS: C_18_H_34_NO_4_S for [M-H], calculated: 360.2, found 360.2.

**1e:** ^1^H NMR (300 MHz, D_2_O): 4.23-2.20 (m, 2H), 3.88-3.84 (dd, *J*=7.5, 4.2 Hz, 1H), 3.71-3.47 (m, 8H), 3.10-2.93 (q, *J*=15, 7.5, 4.2 Hz, 2H), 2.82-2.66 (dd, *J*=8.4, 2.1 Hz, 2H), 2.71-2.66 (dd, *J*=8.4, 2.1 Hz, 2H), 1.10 (t, *J*=6.9 Hz, 3H); ^13^C NMR (75 MHz, D_2_O): 174.28, 172.64, 69.59, 68.82, 68.36, 66.58, 64.03, 53.48, 33.92, 32.08, 26.29, 14.06; M.P.: 195 ^o^C; EI-MS: C_10_H_18_NO_5_S for [M-EtO], calculated: 264.1, found 264.1.

**General procedure for the preparation of cysteine-based DKP small molecules:**

In a two-neck, round-bottom flask, a suspension of cysteine precursor (0.5 g) in 5 mL of 1,2-dichlorobenzene or NMP was stirred at room temperature and purged with nitrogen for 10 min. P_2_O_5_ (0.05 g, 10 wt%) was then added and the mixture was stirred for another 30 min at ambient temperature and then at 85 ^o^C overnight. A sticky compound was obtained after o-dichlorobenzene was removed.

**2a:** Methanol (10 mL) was added into the flask, **2a** was collected as precipitates by suction filtration (88% yield). ^1^H NMR (300 MHz, DMSO-d_6_): (Trans-) 8.26 (s, 1H), 4.22 (s, 1H), 3.60 (s, 3H), 3.08-2.78 (q, *J*=13.8, 4.2, 3.3Hz, 2H), 2.77-2.72 (dd, *J*=8.4, 1.2 Hz, 2H), 2.61-2.56 (dd, *J*=7.8, 0.9 Hz, 2H); (Ci*s-*) 8.20 (s, 1H), 4.15 (s, 1H), 3.60 (s, 3H), 3.02-2.85 (q, *J*=14.1, 4.8, 4.2Hz, 2H), 2.78-2.73 (dd, *J*=8.1, 0.9 Hz, 2H), 2.62-2.57(dd, *J*=7.8, 0.9 Hz, 2H); ^13^C NMR (75 MHz, DMSO-d_6_): (Trans-) 172.30, 166.94, 55.19, 51.84, 36.17, 34.77, 28.11; (Cis*-*) 172.36, 166.47, 55.04, 51.89, 35.64, 34.62, 27.80. ES^+^-MS: C_14_H_22_N_2_O_6_S_2_Na for [M+Na^+^], calculated: 401.1, found 401.1.

**2b:** Cis- and trans- isomers of **2b** were obtained in a total yield of 88% after column chromatography eluting with hexane – acetone (2:1 v/v) and hexane – acetone (1:1 v/v), respectively. ^1^H NMR (300 MHz, DMSO-d_6_): (Trans*-*) 8.25 (s, 1H), 4.22 (s, 1H), 4.02 (t, *J*=6.6Hz, 2H), 3.08-2.79 (q, *J*=13.8, 4.2, 3.3Hz, 2H), 2.76-2.71 (dd, *J*=8.1, 0.9 Hz, 2H), 2.60-2.55 (dd, *J*=6.9, 0.9 Hz 2H), 1.60-1.50 (m, 2H), 1.39-1.26 (tq, *J*=7.5 Hz, 2H), 0.89 (t, *J*=7.2 Hz, 3H); (Cis*-*) 8.20 (s, 1H), 4.14 (s, 1H), 4.02 (t, *J*=6.6 Hz, 2H), 3.02-2.85 (q, *J*=14.1, 4.8, 4.2 Hz, 2H), 2.77-2.71 (dd, *J*=7.2, 0.9 Hz, 2H), 2.60-2.55 (dd, *J*=7.8, 0.9 Hz, 2H), 1.60-1.50 (m, 2H), 1.39-1.27 (tq, *J*=7.5 Hz, 2H), 0.88 (t, *J*=7.2 Hz, 3H); ^13^C NMR (75 MHz, DMSO-d_6_): (Trans*-*) 171.87, 166.94, 64.17, 55.20, 36.17, 34.98, 30.63, 28.20, 19.06, 14.01; (Cis*-*) 171.90, 166.46, 64.17, 55.05, 35.67, 34.80, 30.63, 27.84, 19.06, 14.01. ES^+^-MS: C_20_H_34_N_2_O_6_S_2_Na for [M+Na^+^], calculated: 485.2, found 485.2.

**2c**: Cis- and trans isomer of **2c** were obtained in a total yield of 88% after column chromatography eluting with hexane – acetone (2:1 v/v) and hexane – acetone (1:1 v/v), respectively. ^1^H NMR (300 MHz, DMSO-d_6_): (Cis-) 8.20 (s, 1H), 4.14 (s, 1H), 3.99-3.91 (d, *J*=5.4 Hz, 2H), 3.02-2.85 (q, *J*=14.1, 4.8, 4.2 Hz, 2H), 2.77-2.71 (dd, *J*=11.1, 0.9 Hz, 2H), 2.60-2.56 (dd, q, *J*=6.9, 0.9 Hz, 2H), 1.54-1.50 (m, 1H), 1.35-1.24 (m, 8H), 0.88-0.81 (m, 6H); ^13^C NMR (75 MHz, DMSO-d_6_): (Cis*-*) 171.92, 166.45, 66.51, 55.04, 38.58, 35.68, 34.80, 31.13, 30.21, 28.77, 27.91, 23.65, 22.85, 14.35, 11.24; ES^+^-MS: C_28_H_50_N_2_O_6_S_2_Na for [M+Na^+^], calculated: 597.3, found 597.4.

**2d**: By adding 10 mL of methanol to the residue, **2d** precipitated out and was collected by suction filtration (80% yield). ^1^H NMR (300 MHz, DMSO-d_6_): (*Cis-*) 8.21 (s, 1H), 4.14 (s, 1H), 4.00 (t, *J*=6.6Hz, 2H), 3.02-2.84 (q, *J*=14.1, 4.8, 4.2 Hz, 2H), 2.77-2.72 (dd, *J*=6.9, 0.9Hz, 2H), 2.59-2.55 (dd, *J*=6.9, 0.9Hz, 2H), 1.55-1.54 (m, 2H), 1.24 (s, 18H), 0.85 (t, *J*=6.6Hz, 3H); ^13^C NMR (75 MHz, DMSO-d_6_): (Cis*-*) 171.87, 166.42, 64.46, 55.08, 35.74, 34.81, 31.77, 29.48, 29.18, 29.12, 28.57, 27.91, 25.81, 22.56, 14.41; (Trans*-*) 171.86, 166.93, 64.46, 55.21, 36.17, 34.97, 31.77, 28.57, 25.82, 22.57, 14.42. ES^+^-MS: C_36_H_66_N_2_O_6_S_2_Na for [M+Na^+^], calculated: 709.4, found 709.5.

**2e**: After addition of 10 mL of methanol into the flask, **2e** was collected as precipitates by suction filtration (82% yield). ^1^H NMR (300 MHz, DMSO-d_6_): (Cis*-*) 8.20 (s, 1H), 4.13 (m, 3H), 3.61-3.39 (m, 8H), 3.02-2.86 (q, *J*=14.1, 4.8, 4.2 Hz, 2H), 2.77-2.71 (dd, *J*=7.2, 0.9Hz, 2H), 2.63-2.58 (dd, *J*=6.9, 0.9Hz, 2H), 1.10 (t, *J*=7.2Hz, 3H); (Trans*-*) 8.25 (s, 1H), 4.23 (s, 1H), 4.15-4.12 (m, 2H), 3.61-3.39 (m, 8H), 3.08-2.79 (q, *J*=13.8, 4.2, 3.3Hz, 2H), 2.76-2.72 (dd, *J*=8.1, 0.9Hz, 2H), 2.62-2.57 (dd, *J*=8.1, 0.9Hz, 2H). 1.10 (t, *J*=7.2Hz, 3H); ^13^C NMR (75 MHz, DMSO-d_6_): (Cis-)*:* 171.91, 166.47, 70.28, 79.62, 68.69, 66.02, 63.89, 55.02, 35.67, 34.72, 27.74, 15.56; (Trans-): 171.85, 166.93, 70.28, 69.62, 68.69, 66.01, 63.89, 55.19, 36.18, 34.91, 28.08, 15.58. ES^+^-MS: C_24_H_42_N_2_O_10_S_2_Na for [M+Na^+^], calculated: 605.2, found 605.3.

### 2.5.4 Preparation of detection solutions

Stock solutions (0.2 M) of different metal ions, including Pb^2+^, Co^2+^, Ni^+^, Ca^2+^, Mg^2+^, Hg^2+^, Mn^2+^, Zn^2+^, Cu^2+^, Na^+^, Li^+^, Ba^2+^, K^+^ and Ag^+^, were prepared in de-ionized water. The detection solution was prepared by adding 0.01 mL of metal ion solution to 4 mL of **2a** (0.0005 M), respectively.

# Figure S1. CD spectra of L-cysteine and 1a.

# Figure S2. CD spectra upon addition of different metal ions: Zn^2+^, Pd^2+^, Ni^2+^, Mg^2+^, Ca^2+^, Ba^2+^, Co^2+^, Li^+^, Mn^2+^, Pb^2+^ and Na^+^, into cis 2a at 1:1 molar ratio.

# Figure S3. CD spectraof cis 2a upon addition of Cu^2+^ at different molar ratio.

# Figure S4. CD spectraof cis 2a upon addition of Ag^+^ at different molar ratio.

# ^1^H NMR spectrum of 1a in D_2_O


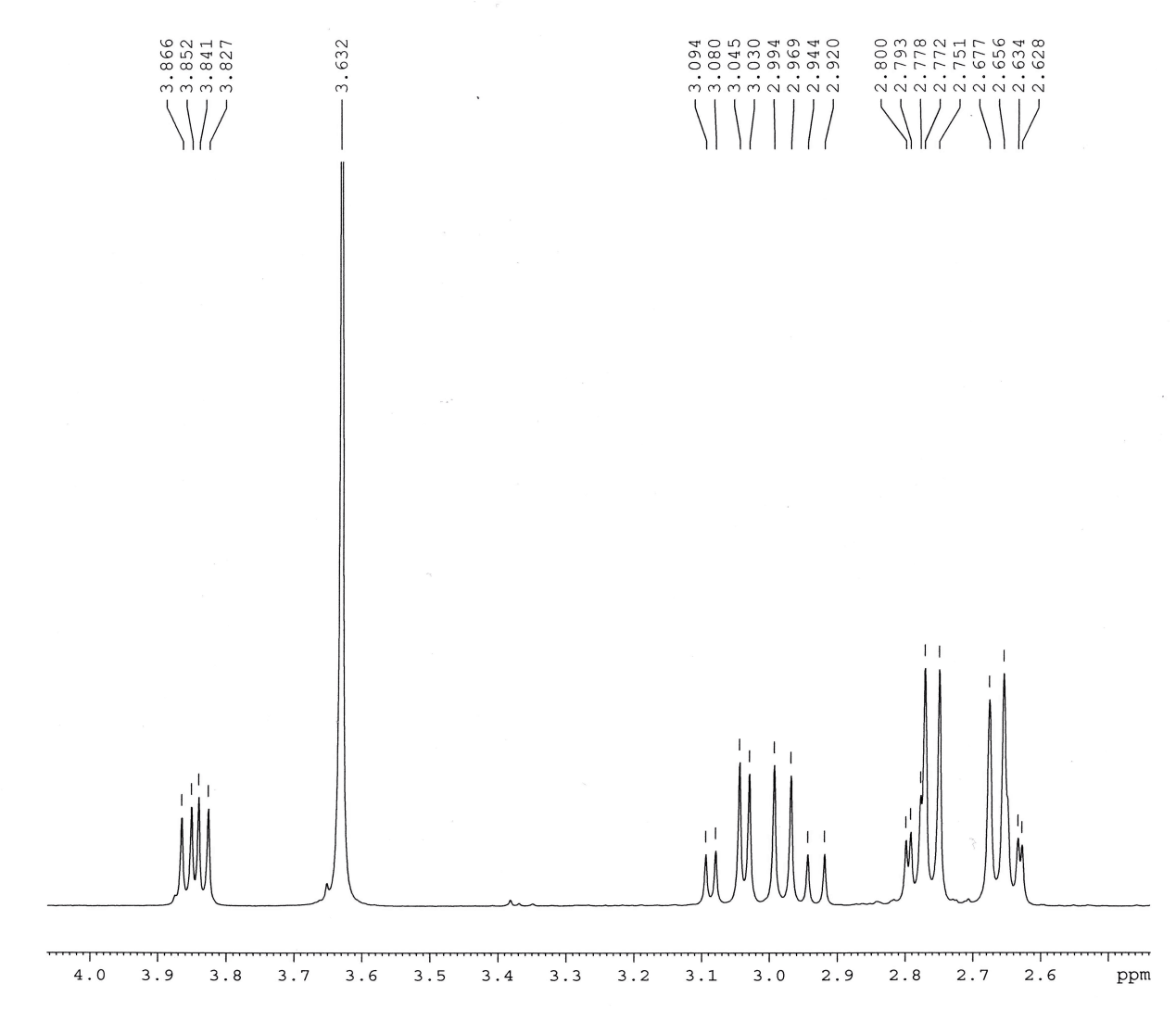


**^13^C NMR spectrum of 1a in D_2_O**


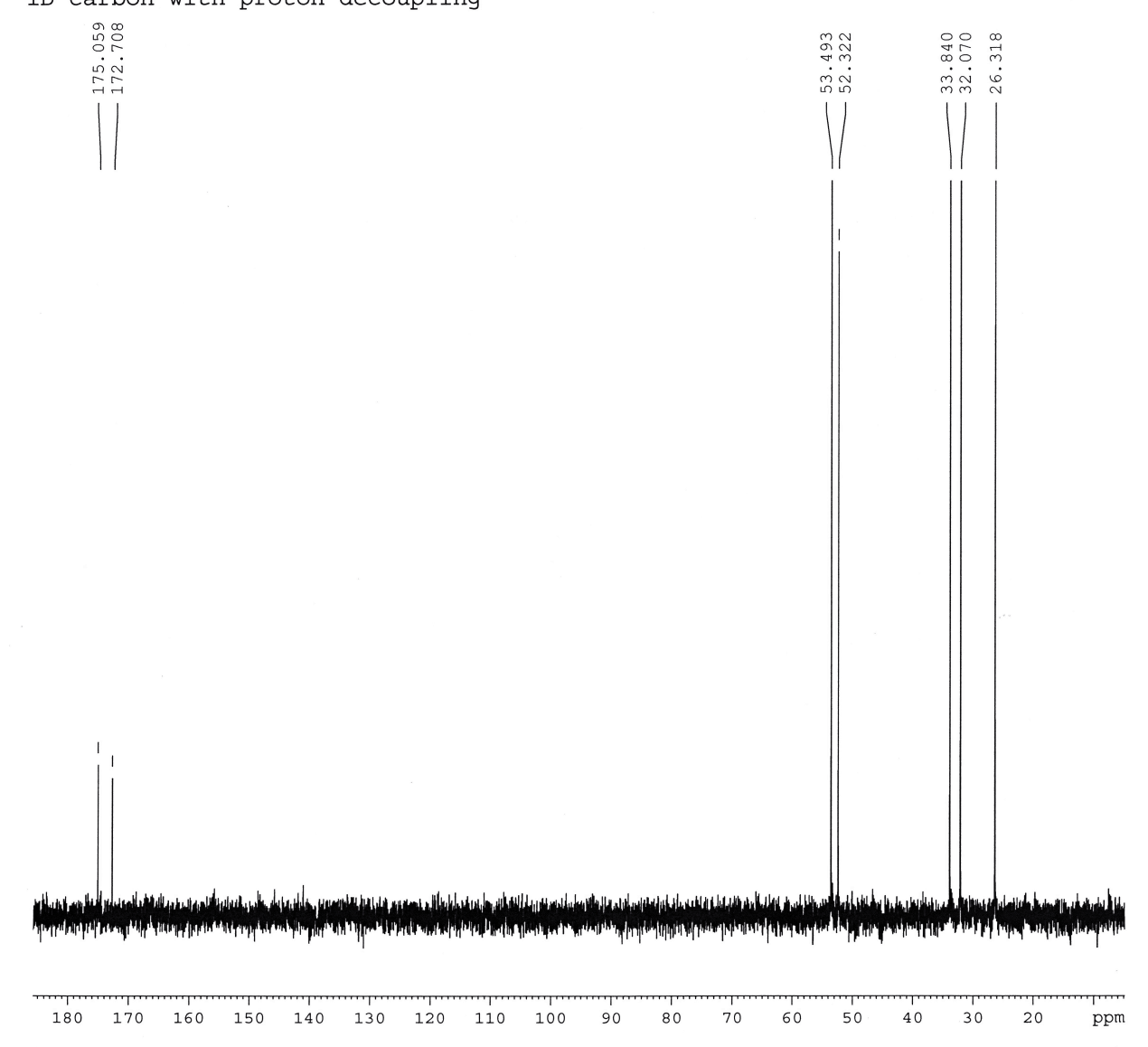


**^1^H NMR spectrum of cis 2a in DMSO-d_6_**


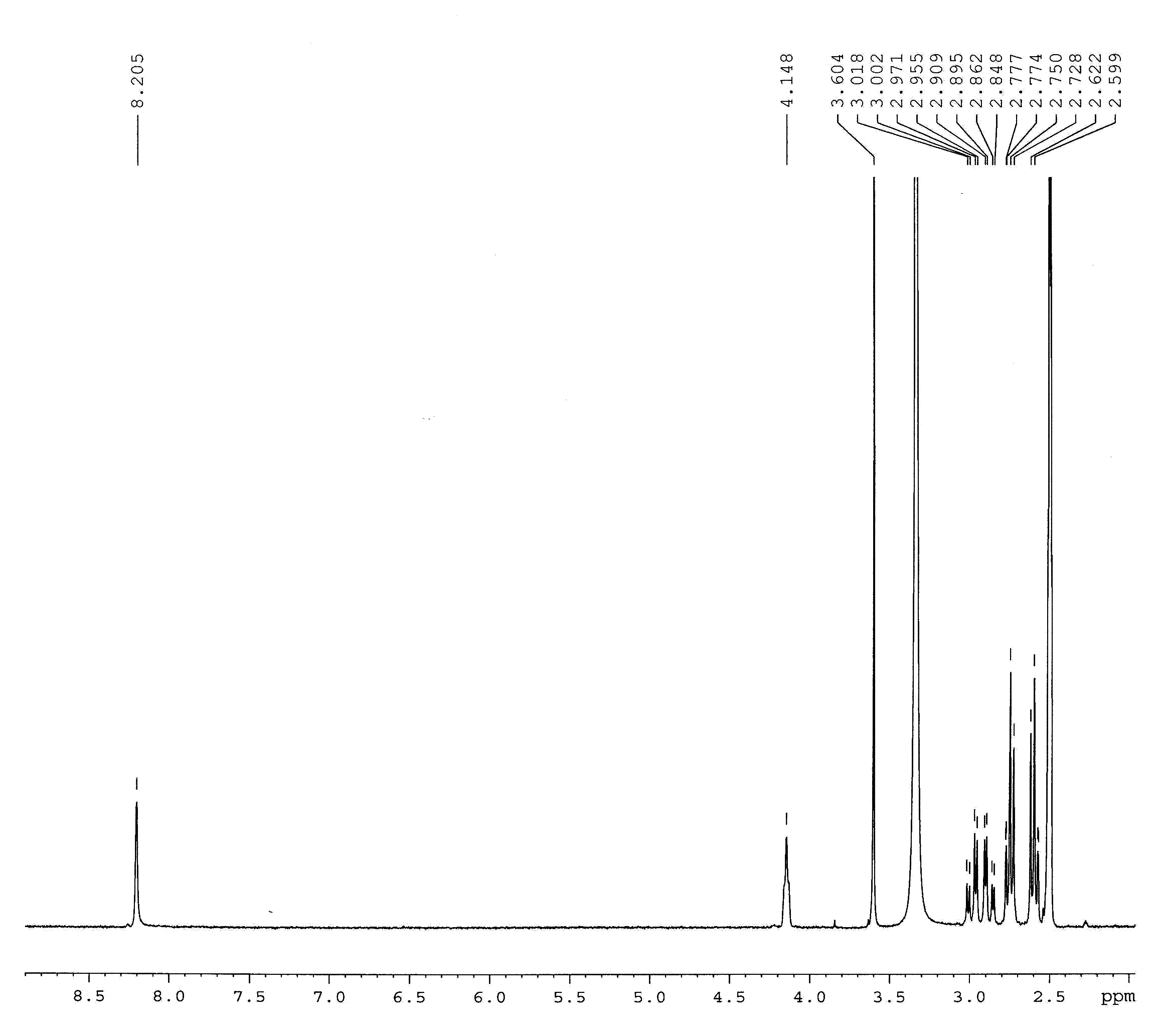


**^13^CNMR spectrum of cis 2a in DMSO-d_6_**


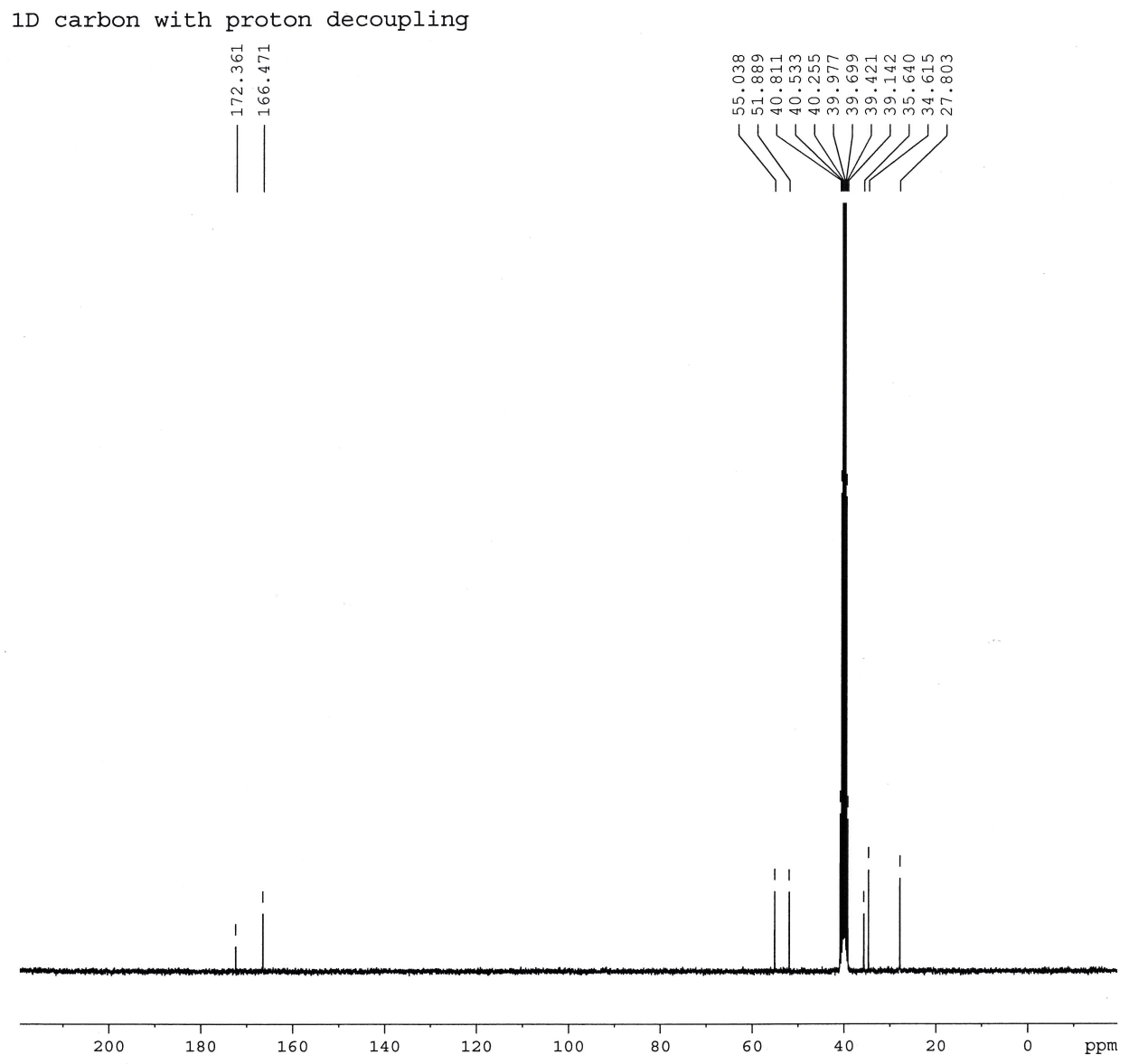


**NOESY of cis 2a in DMSO-d_6_**


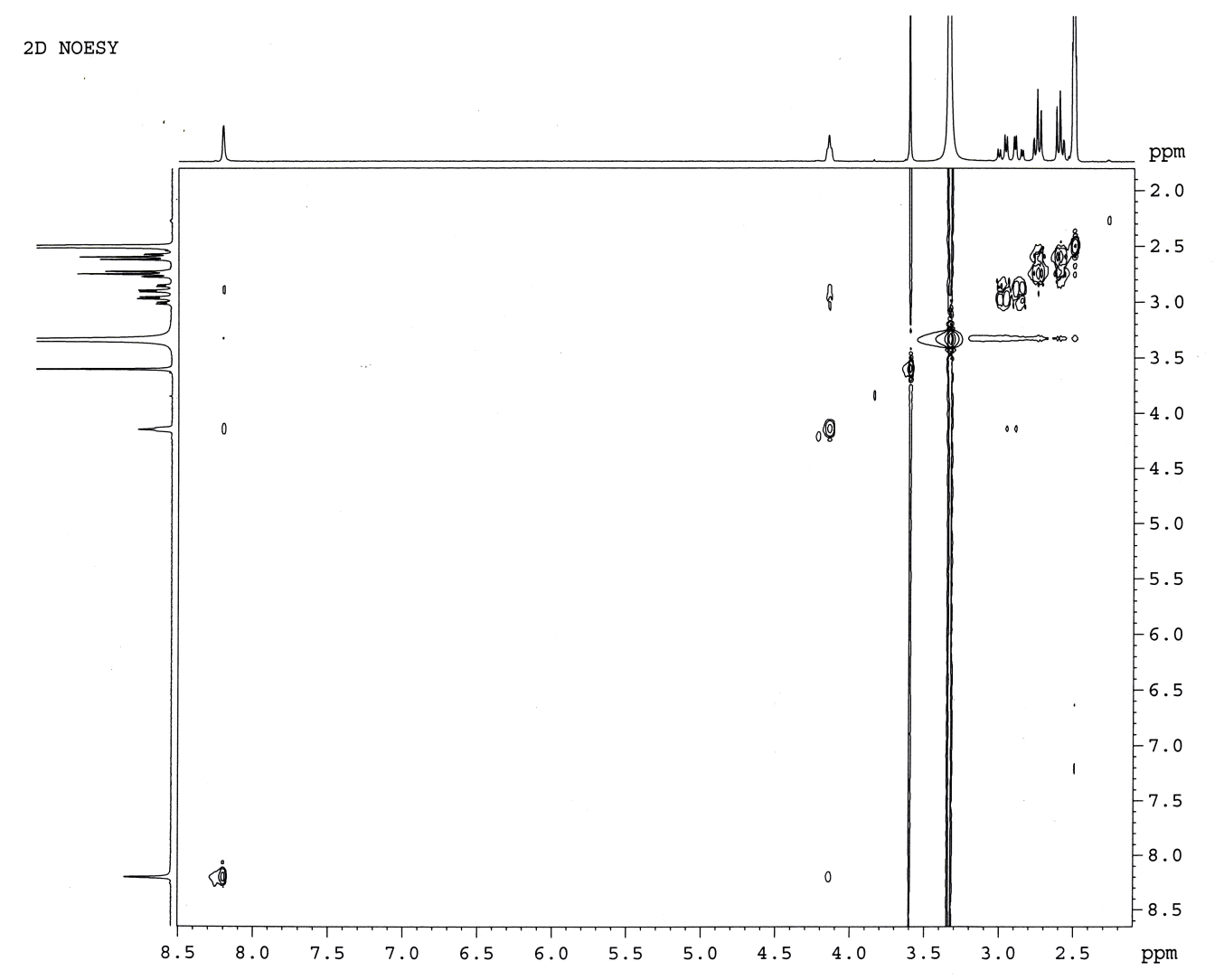


**HSQC of cis 2a in DMSO-d_6_**


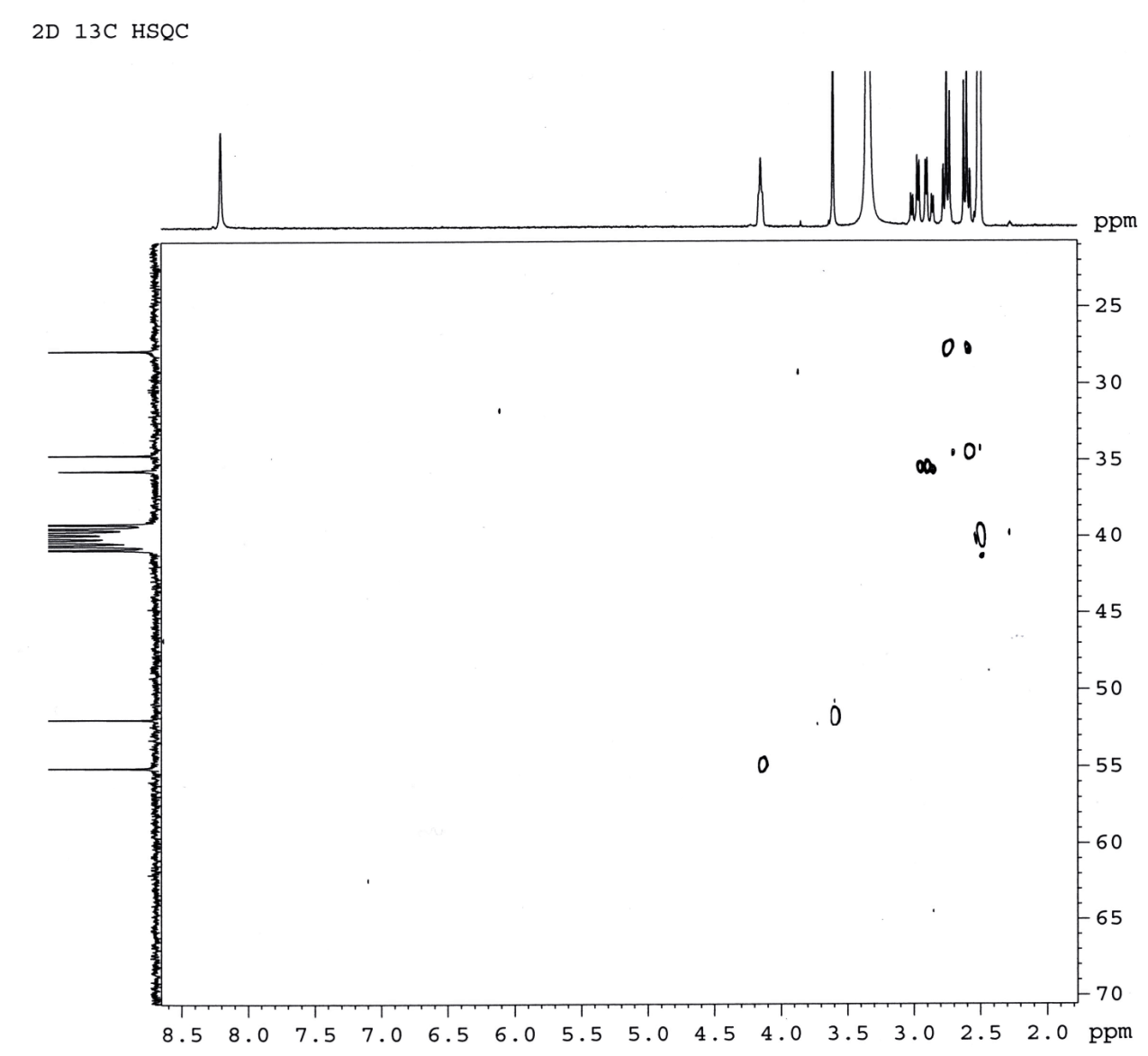


**COSY of cis 2a in DMSO-d_6_**


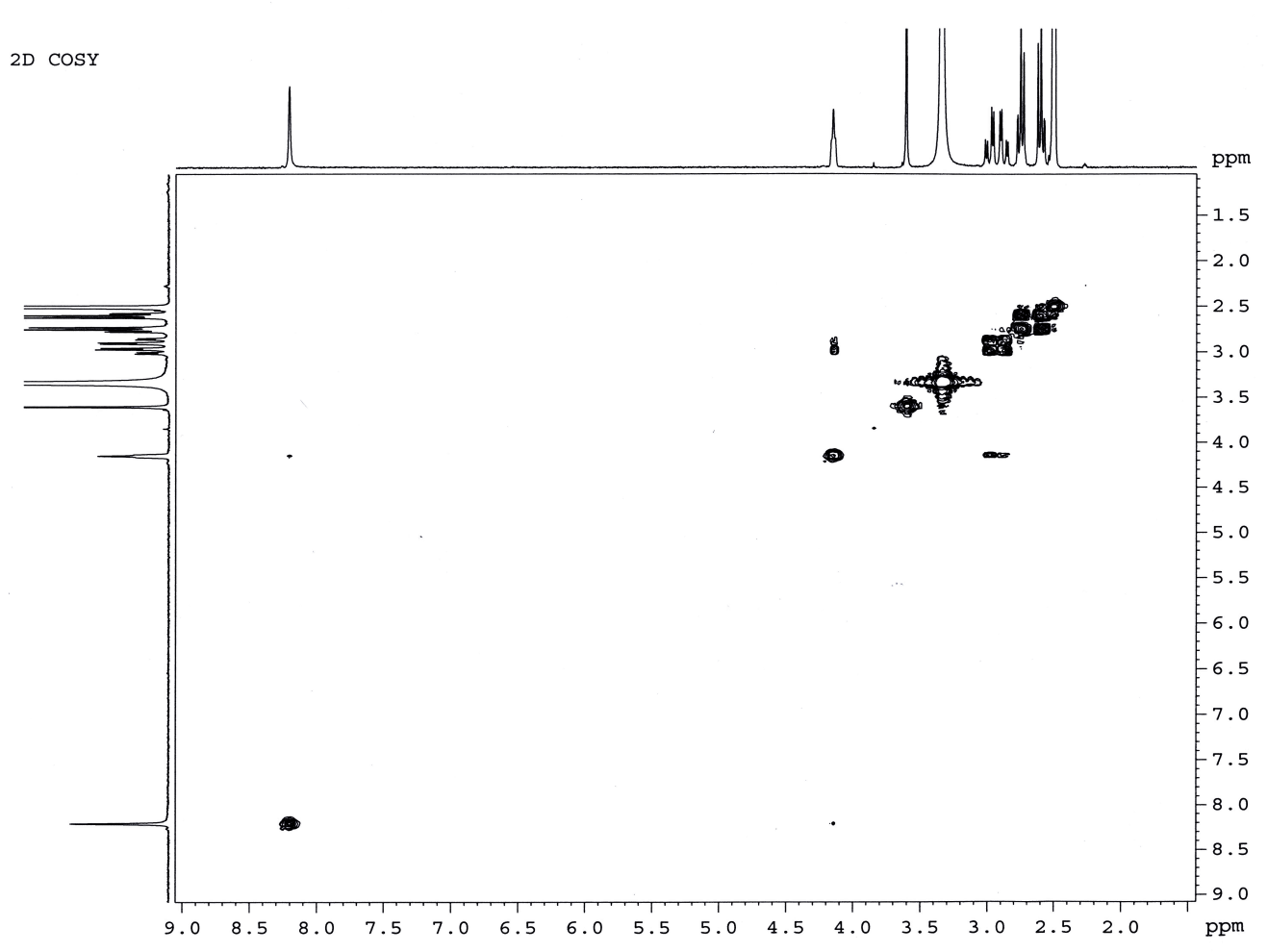


**1D NOE difference of cis 2a in DMSO-d_6_**


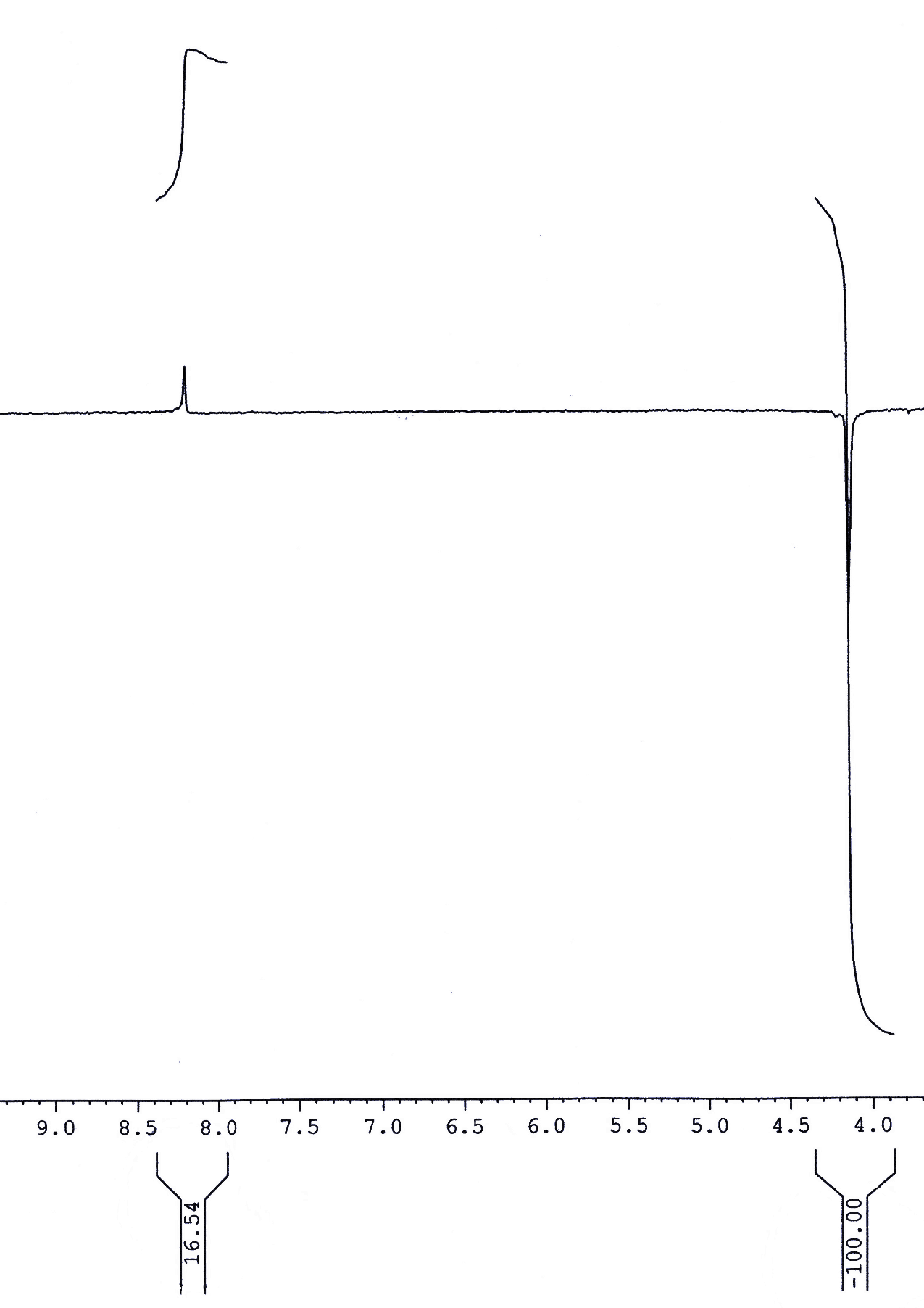


**MS of cis 2a**

**^1^H NMR spectrum of trans 2a in DMSO-d_6_**


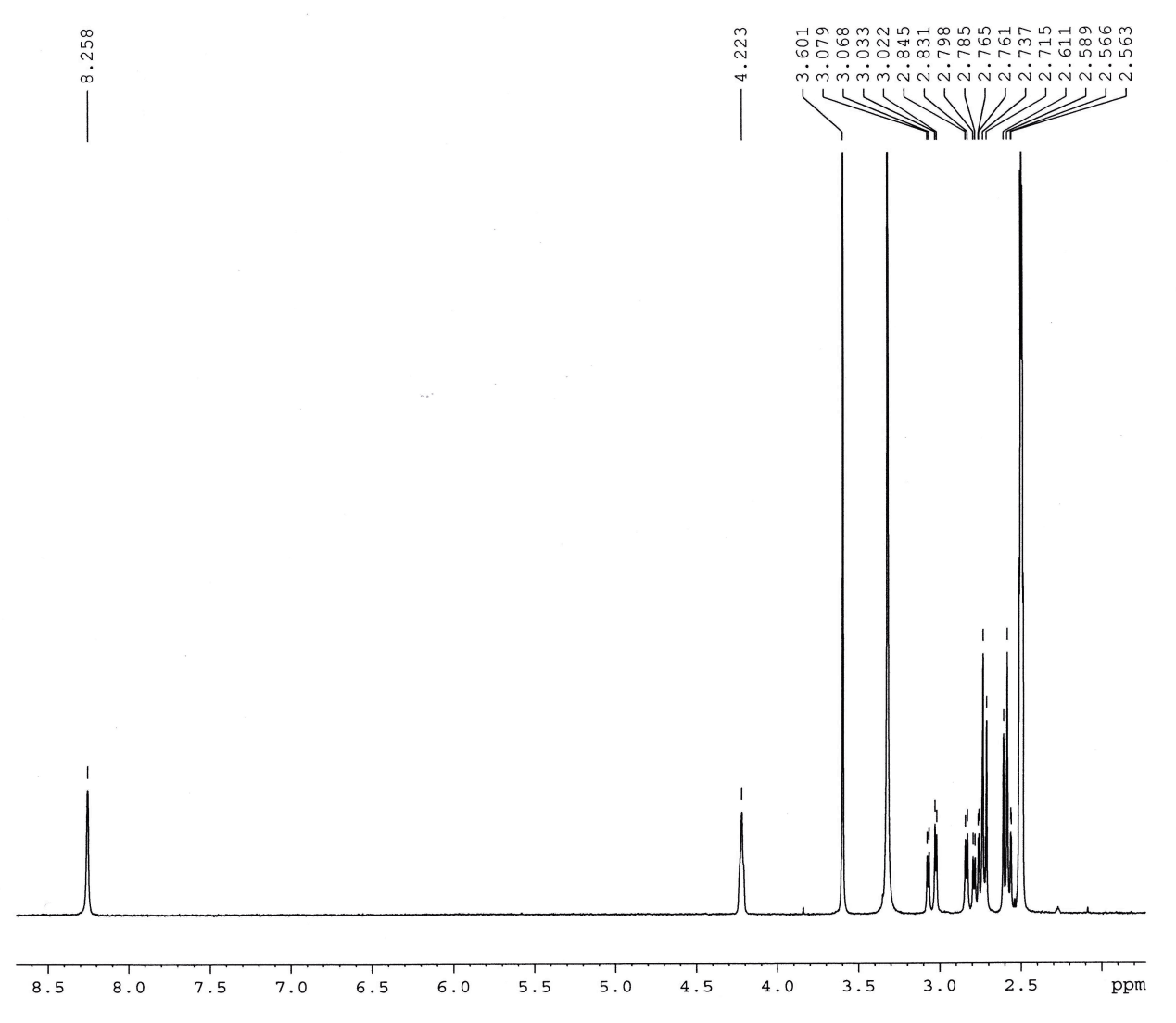


**^13^C NMR spectrum of trans 2a in DMSO-d_6_**


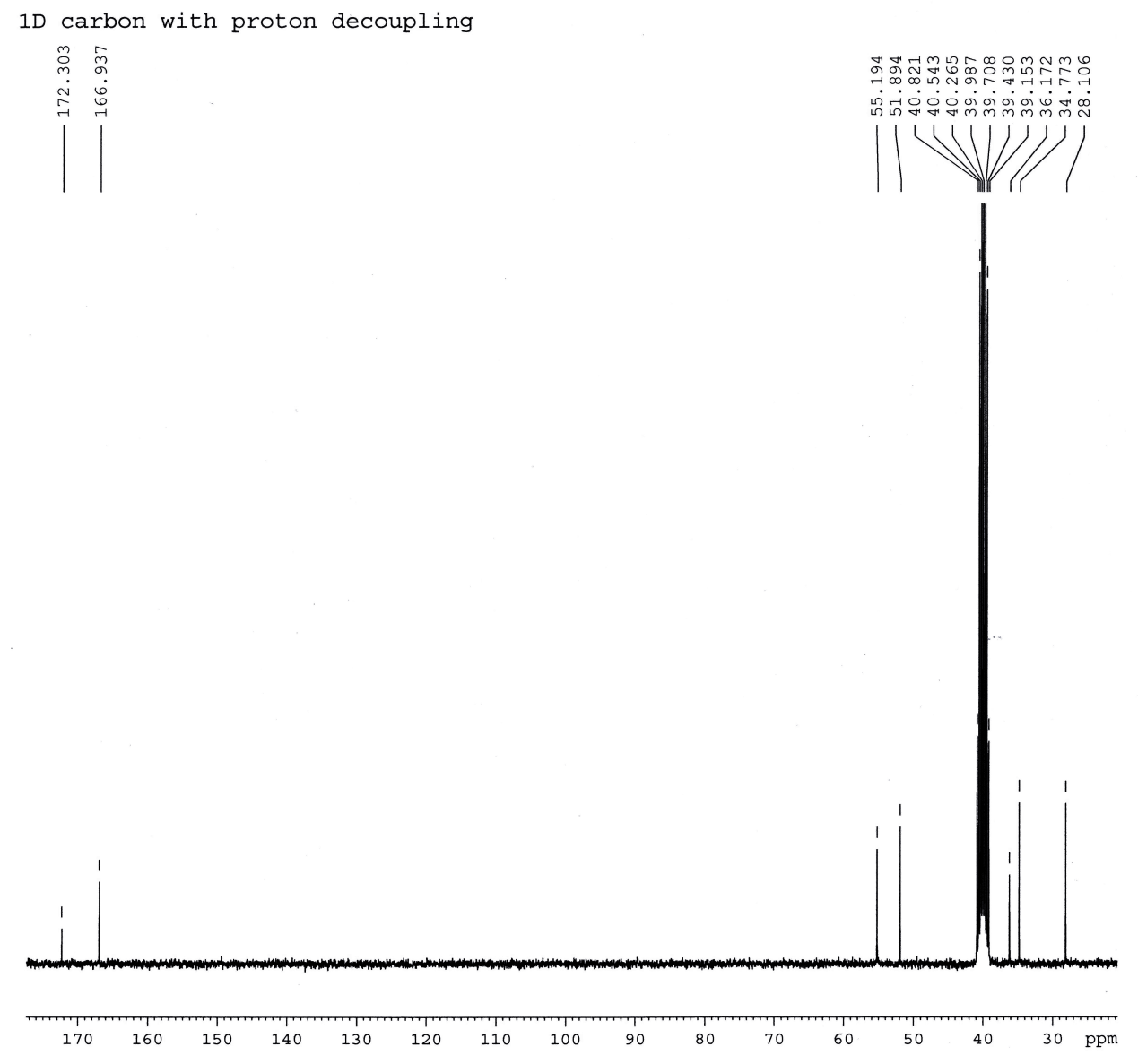
**NOESY of trans 2a in DMSO-d_6_**


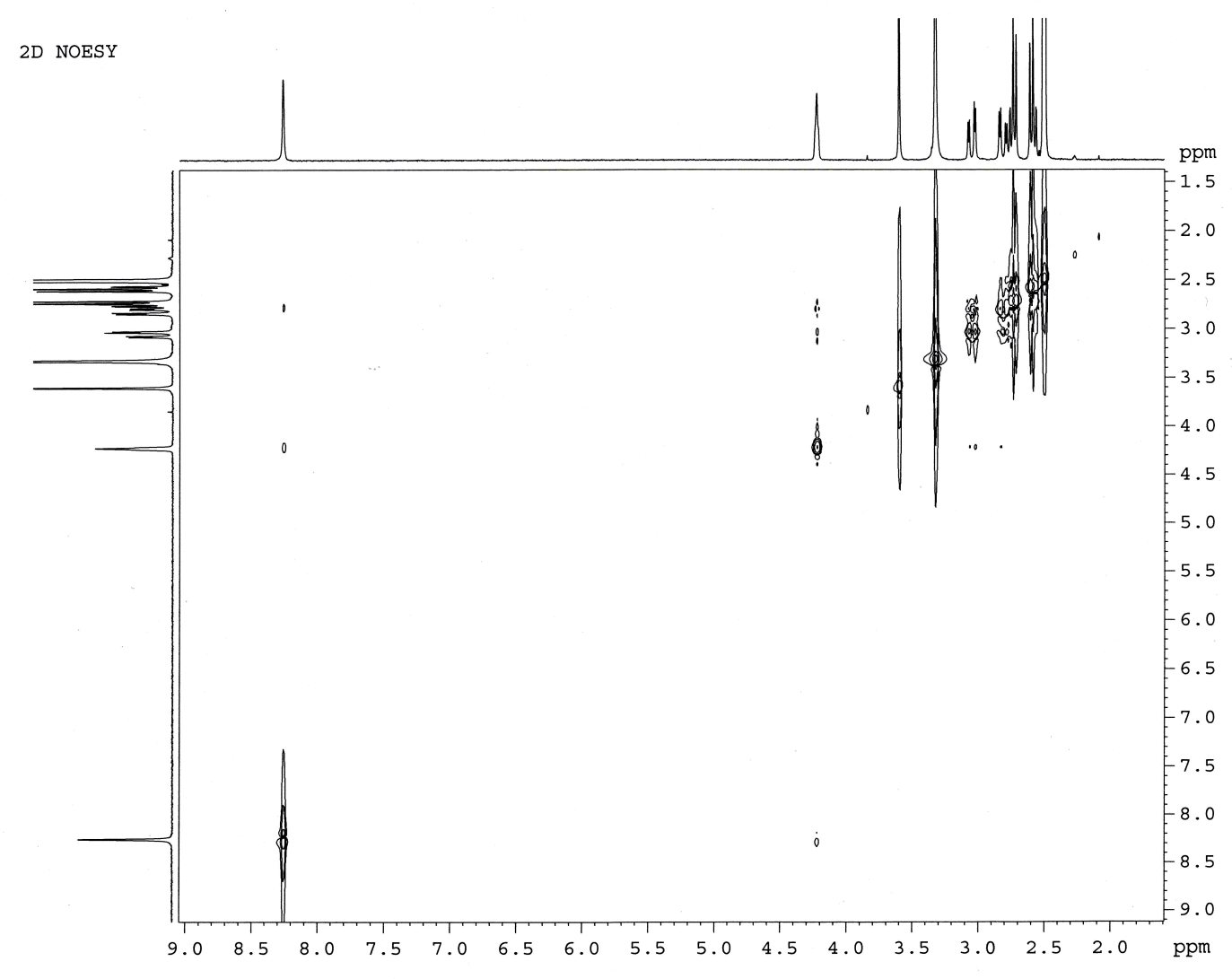


**HSQC of trans 2a in DMSO-d_6_**


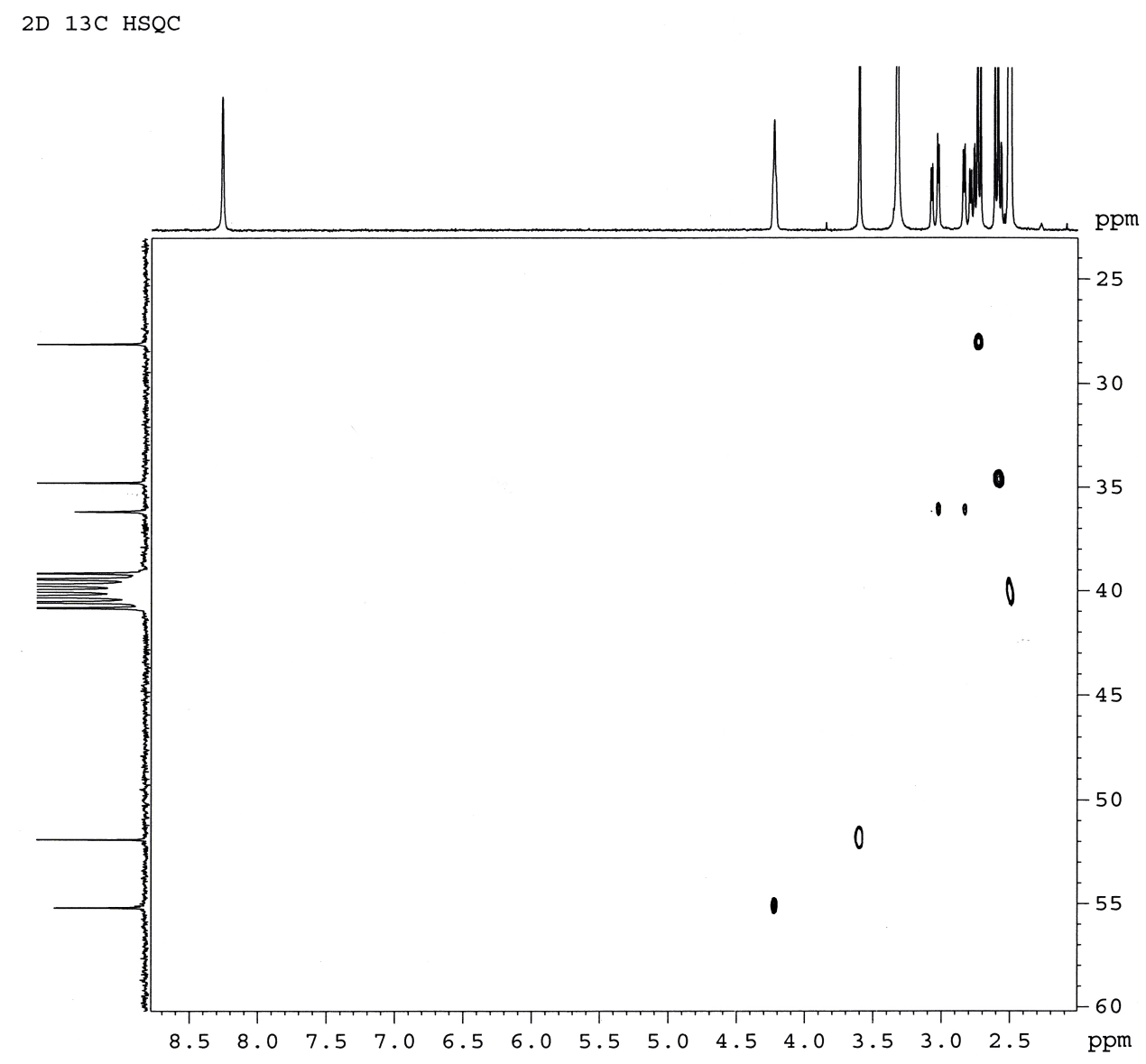
**COSY of trans 2a in DMSO-d_6_**

**
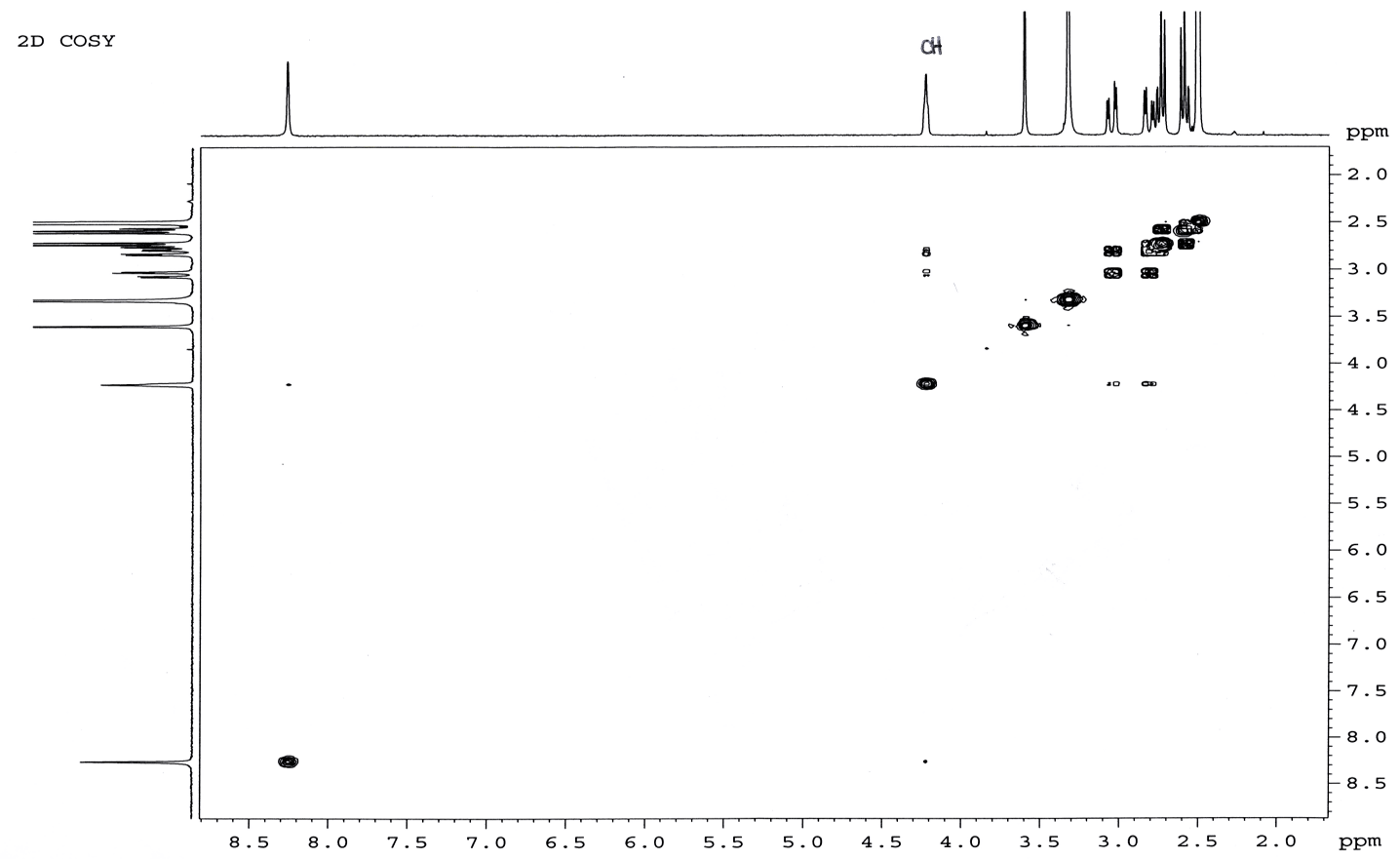
**

**1D NOE difference of trans 2a in DMSO-d_6_**


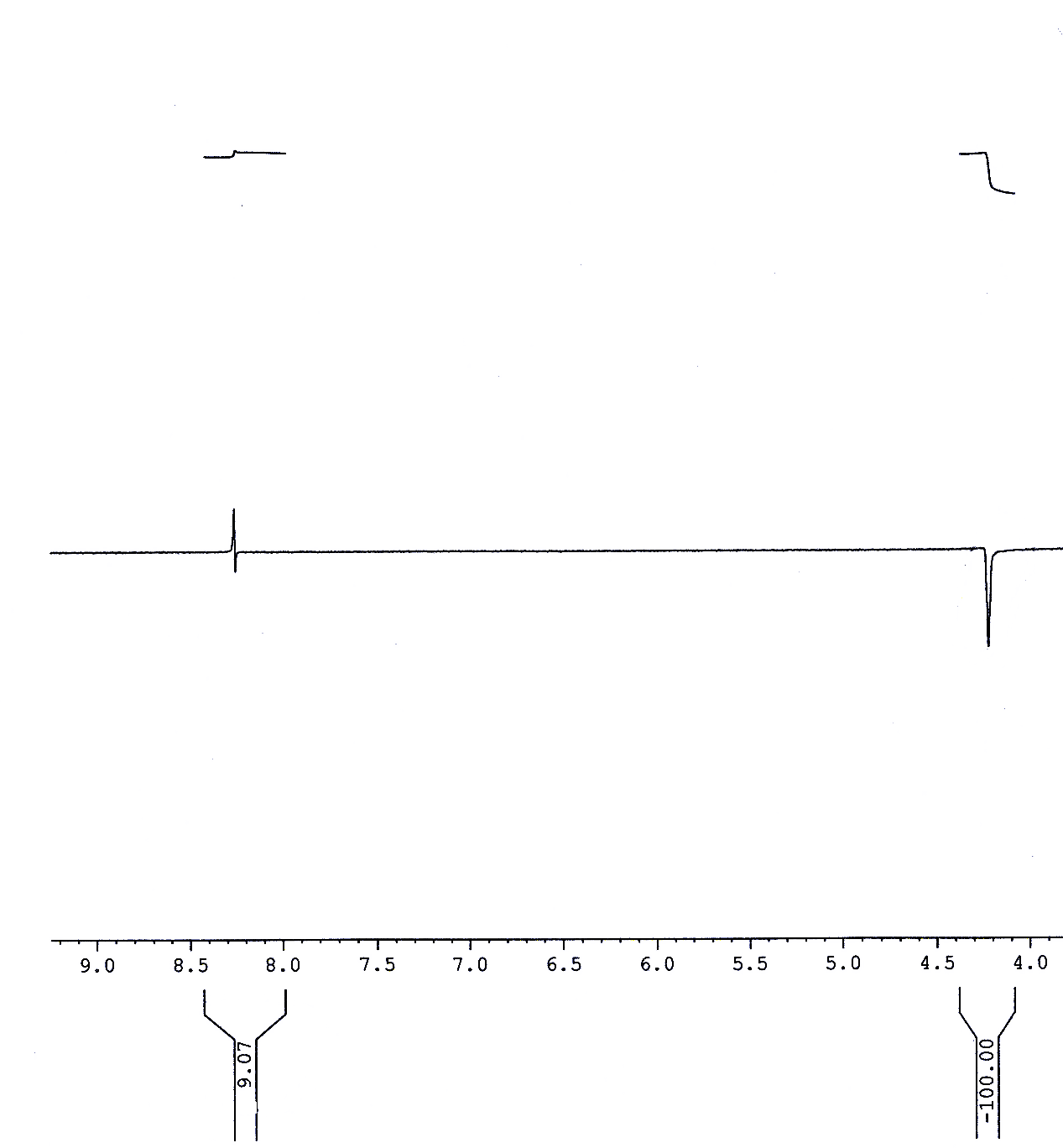


**MS of trans 2a**

# ^1^H NMR spectrum of 1b in D_2_O
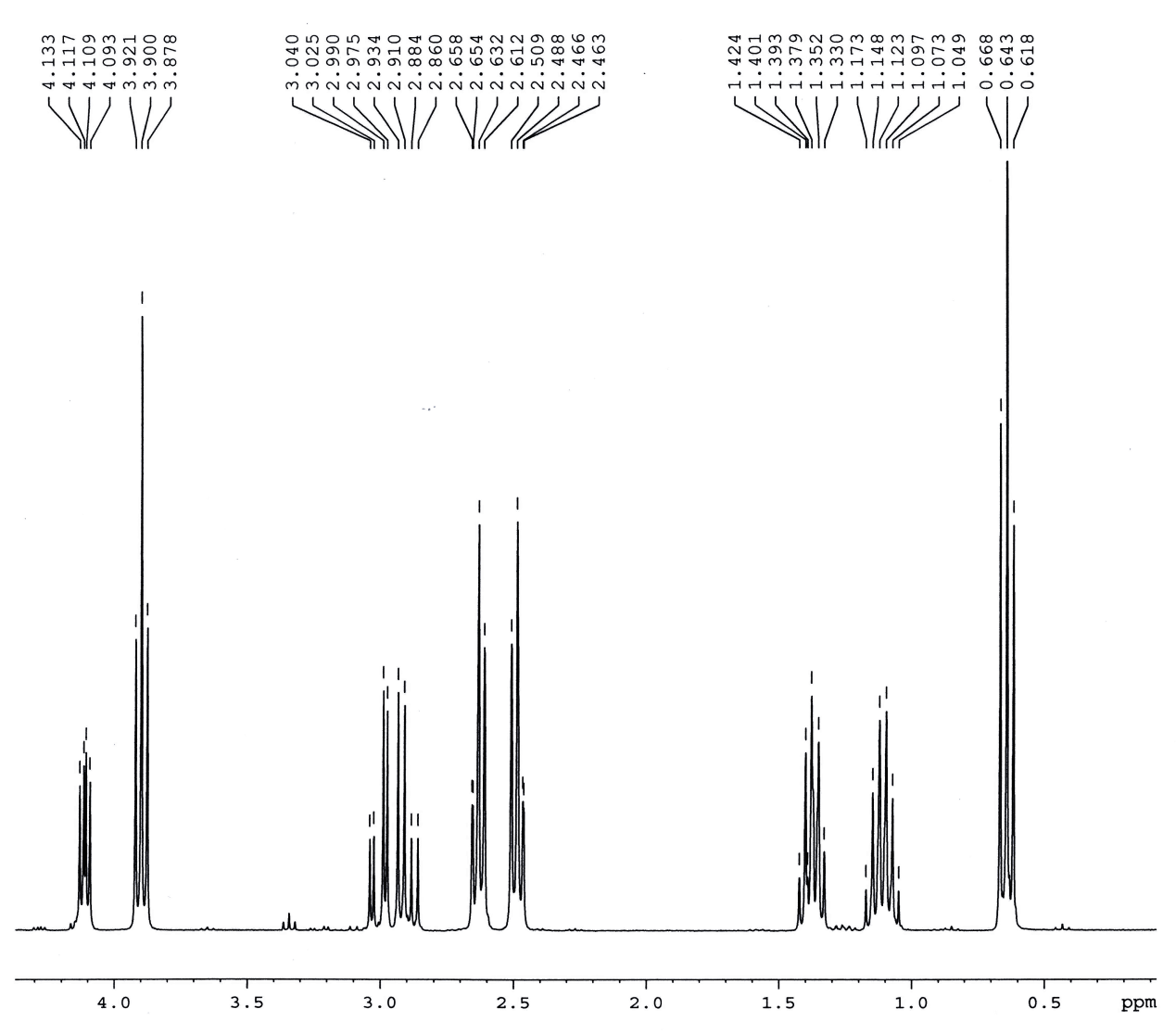


**^13^C NMR spectrum of 1b in D_2_O**


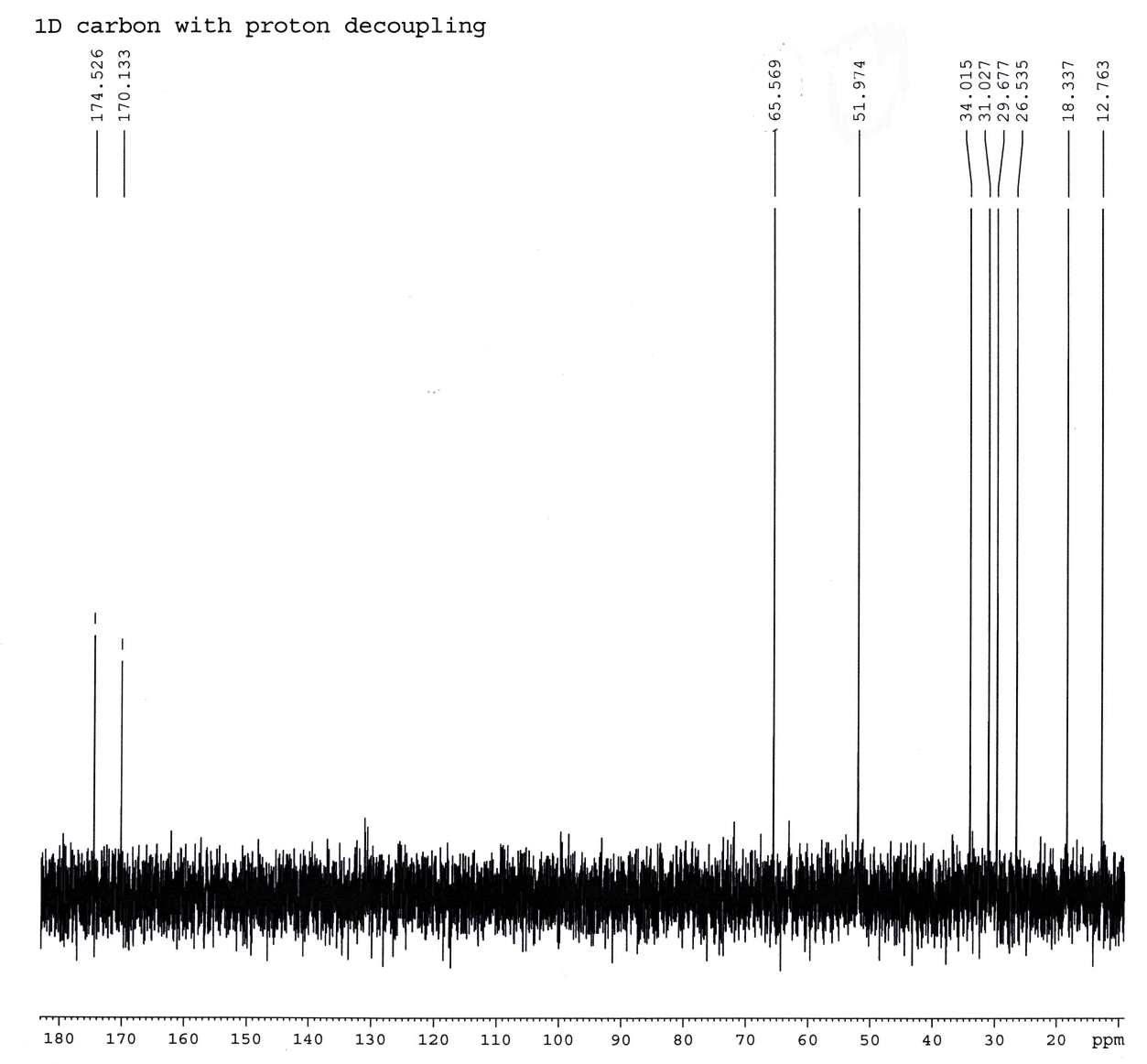


**^1^H NMR spectrum of cis and trans mixture of 2b in DMSO-d_6_**

**
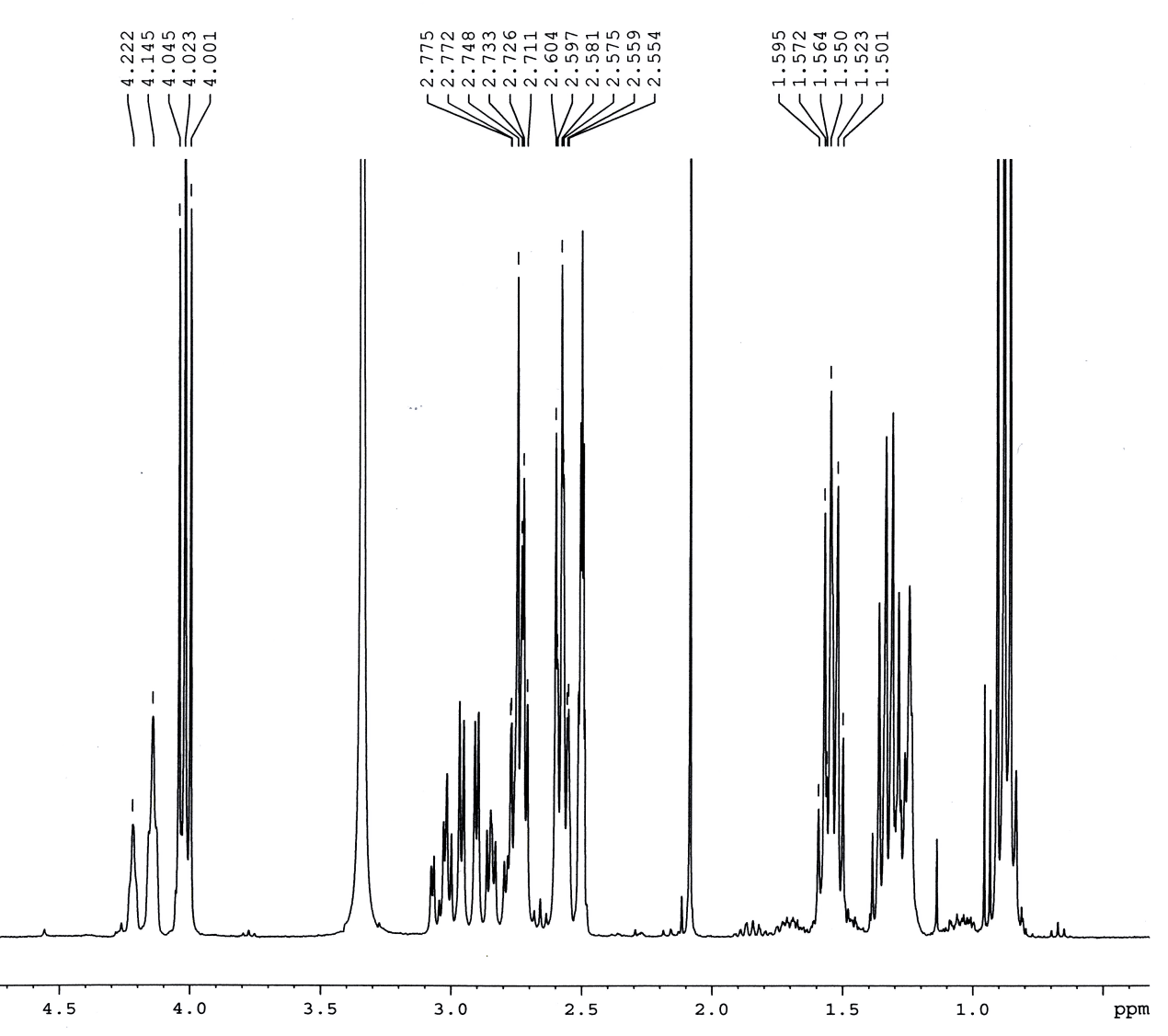
**

**^13^C NMR spectrum of cis and trans mixture of 2b in DMSO-d_6_**


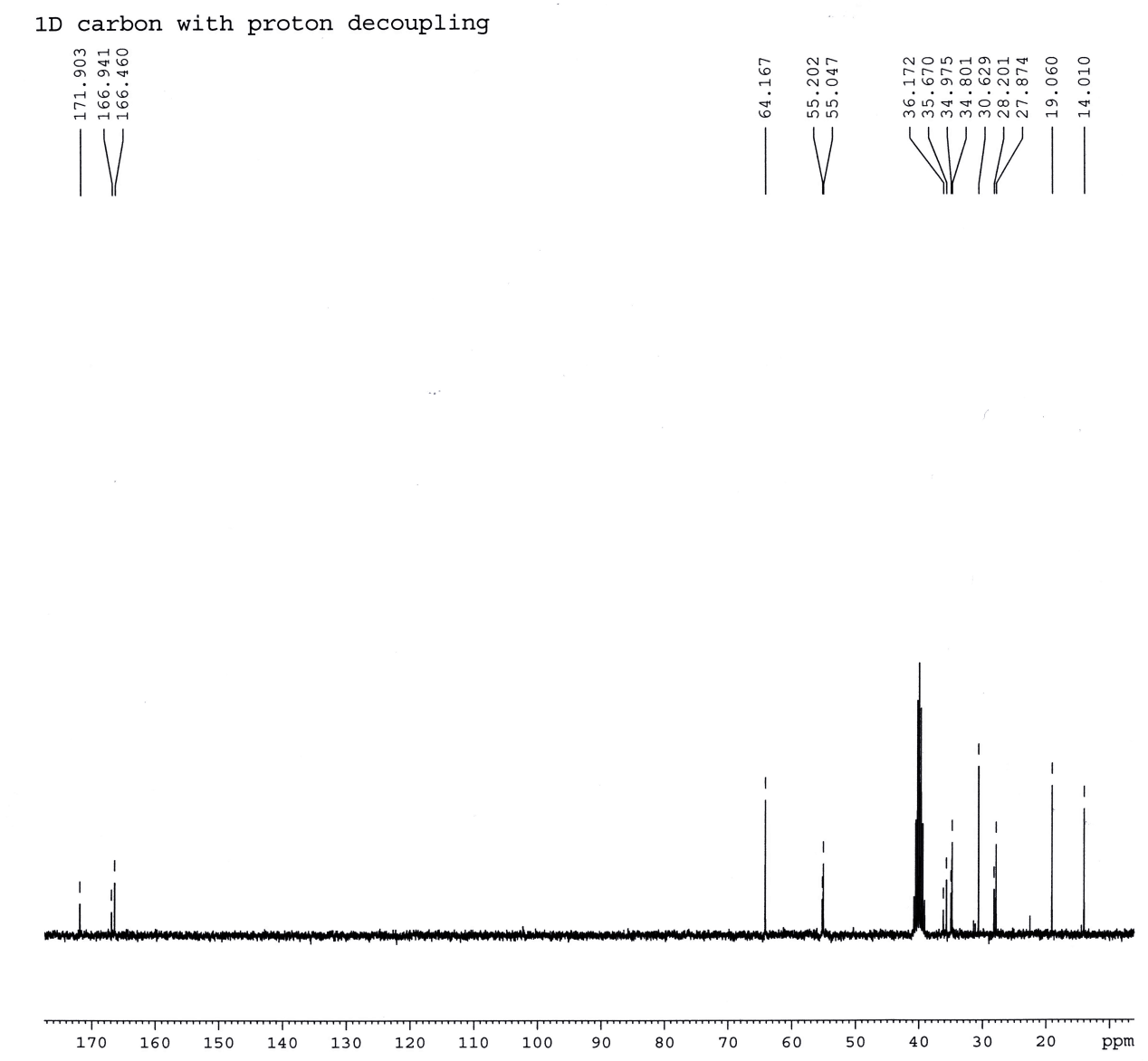


**^1^H NMR spectrum of cis 2b in DMSO-d_6_**

**
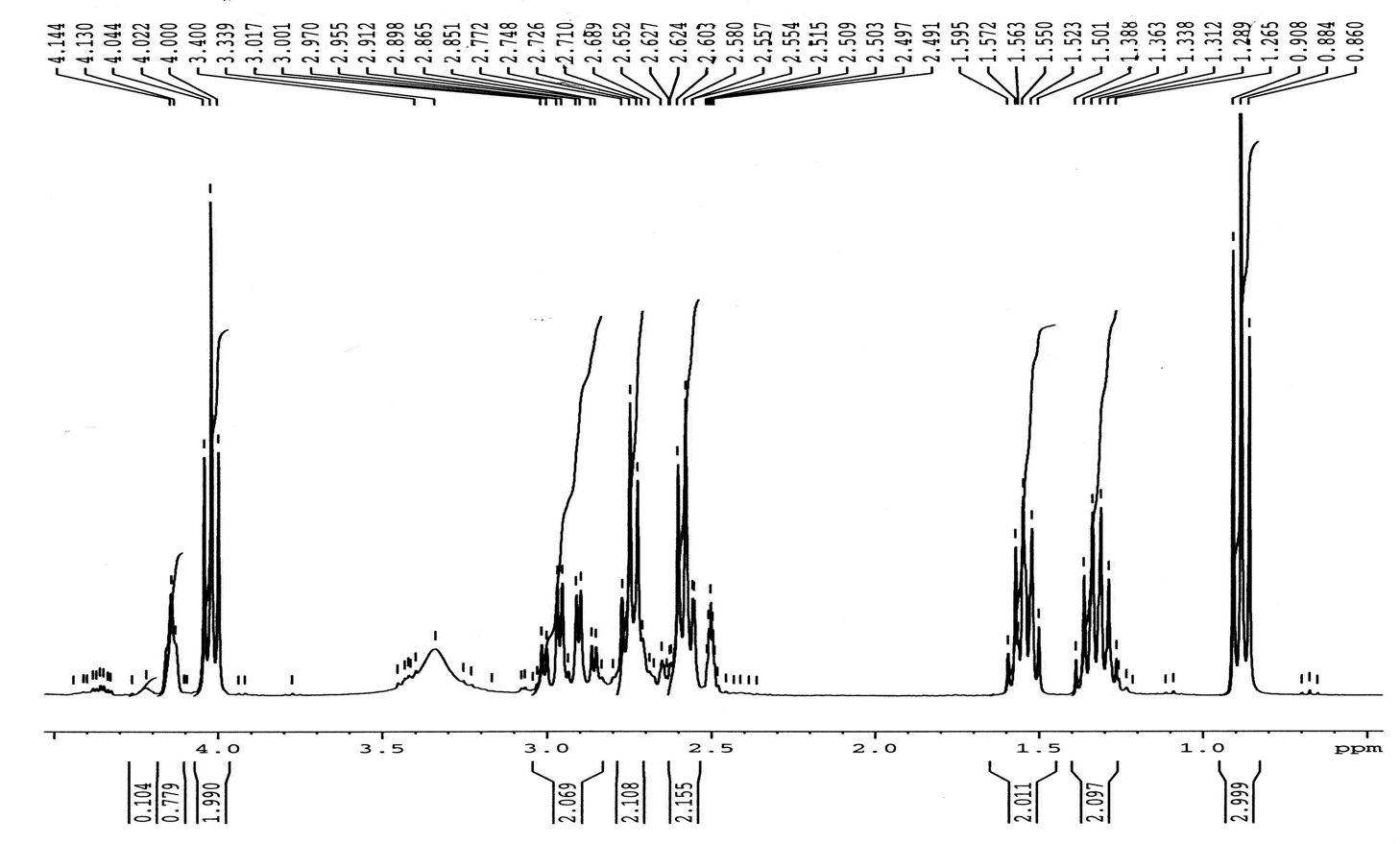
^1^H NMR spectrum of trans 2b in DMSO-d_6_**


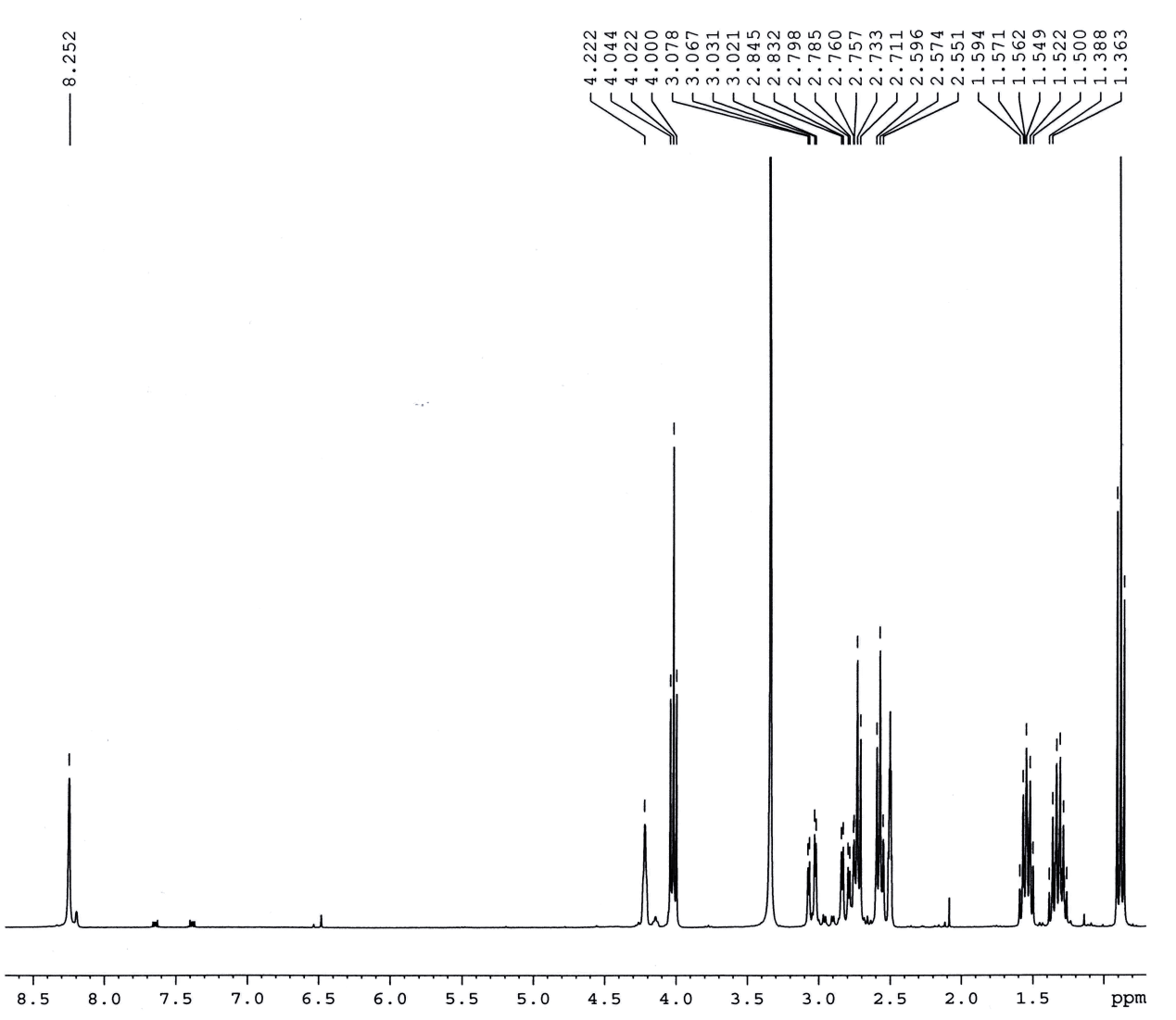


**^13^C NMR spectrum of trans 2b in DMSO-d_6_**


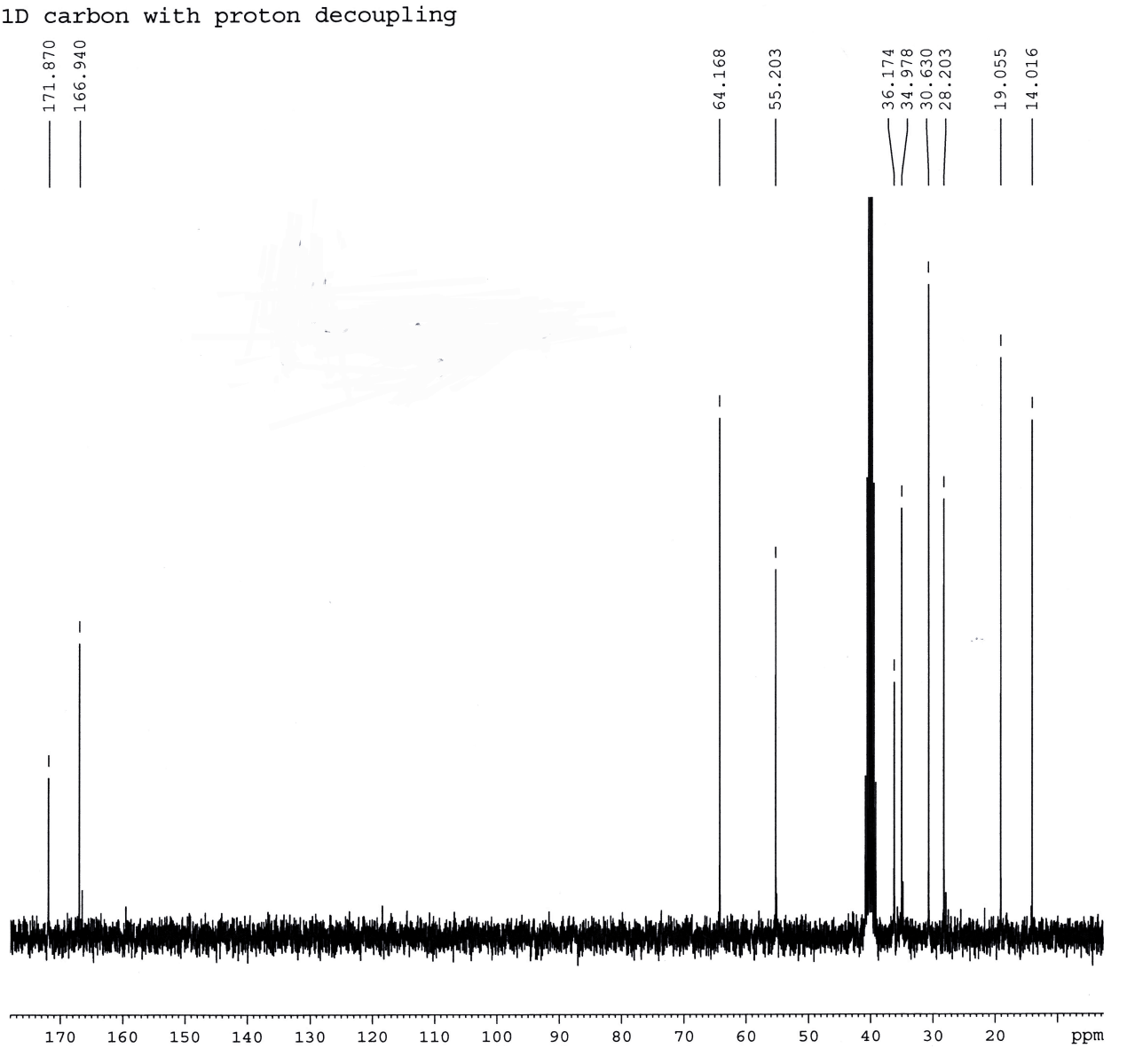


**MS of 2b**

# ^1^H NMR spectrum of 1c in DMSO-d_6_
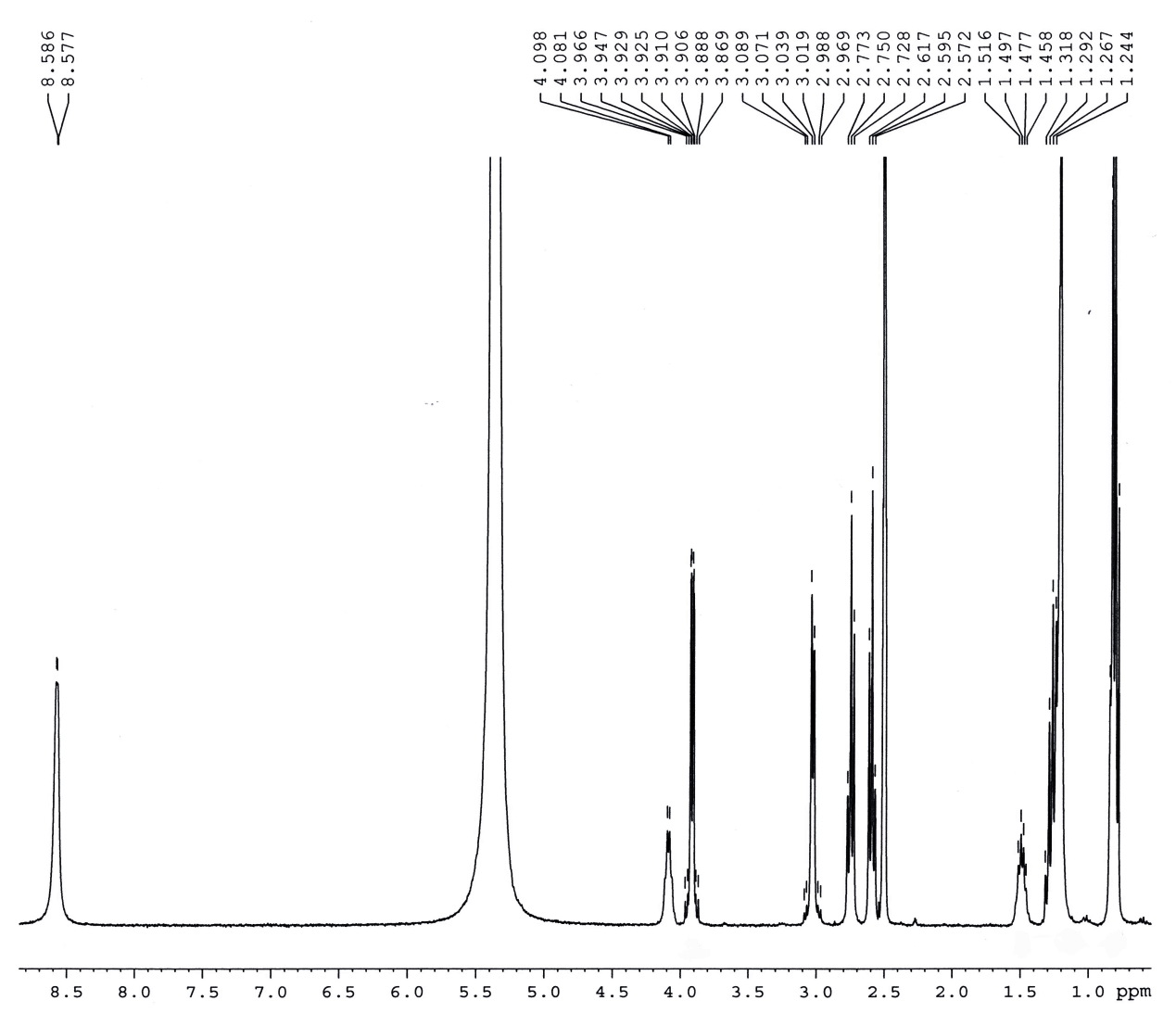


**^13^C NMR spectrum of 1c in DMSO-d_6_**


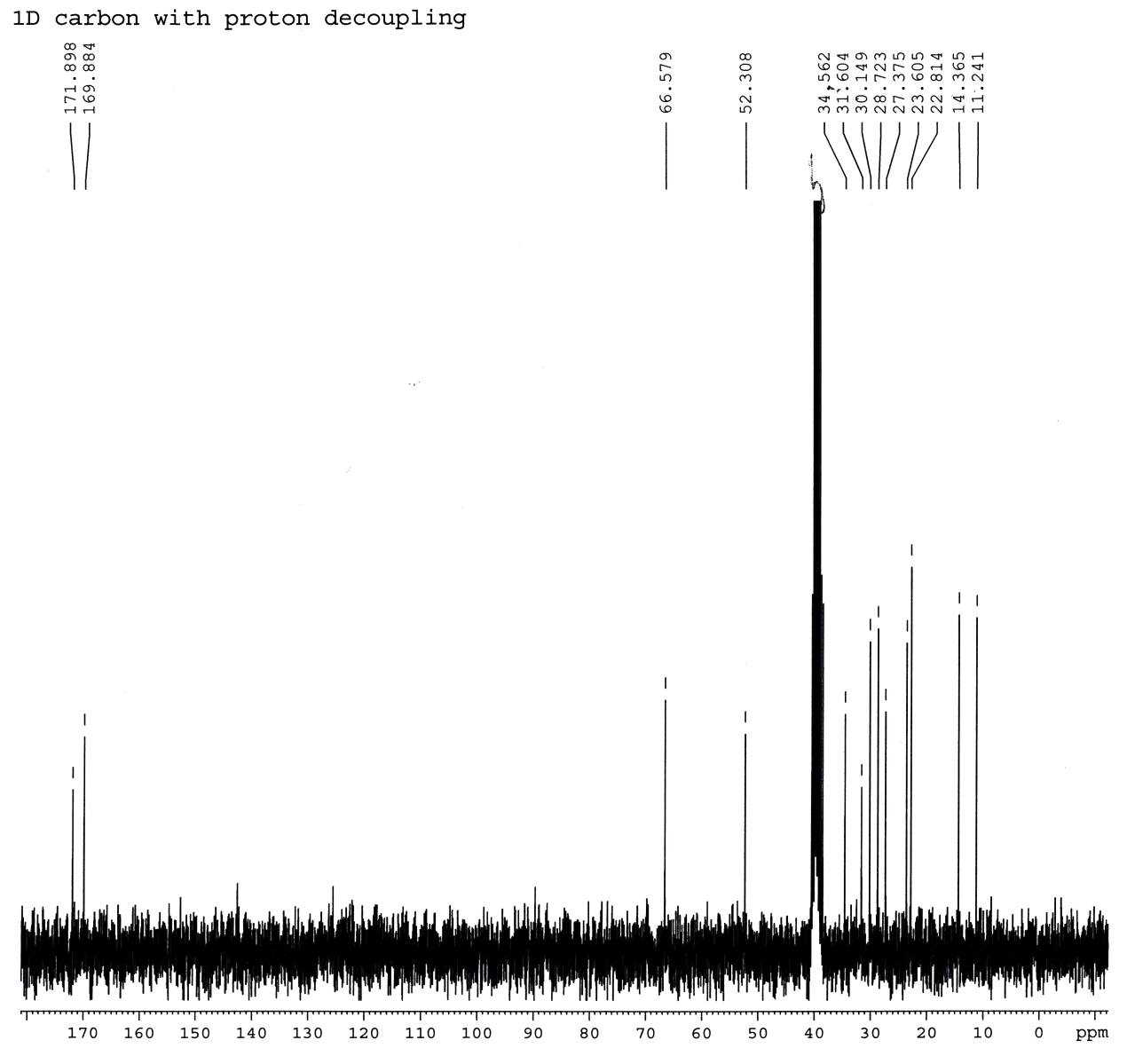


**MS of 1c**

**^
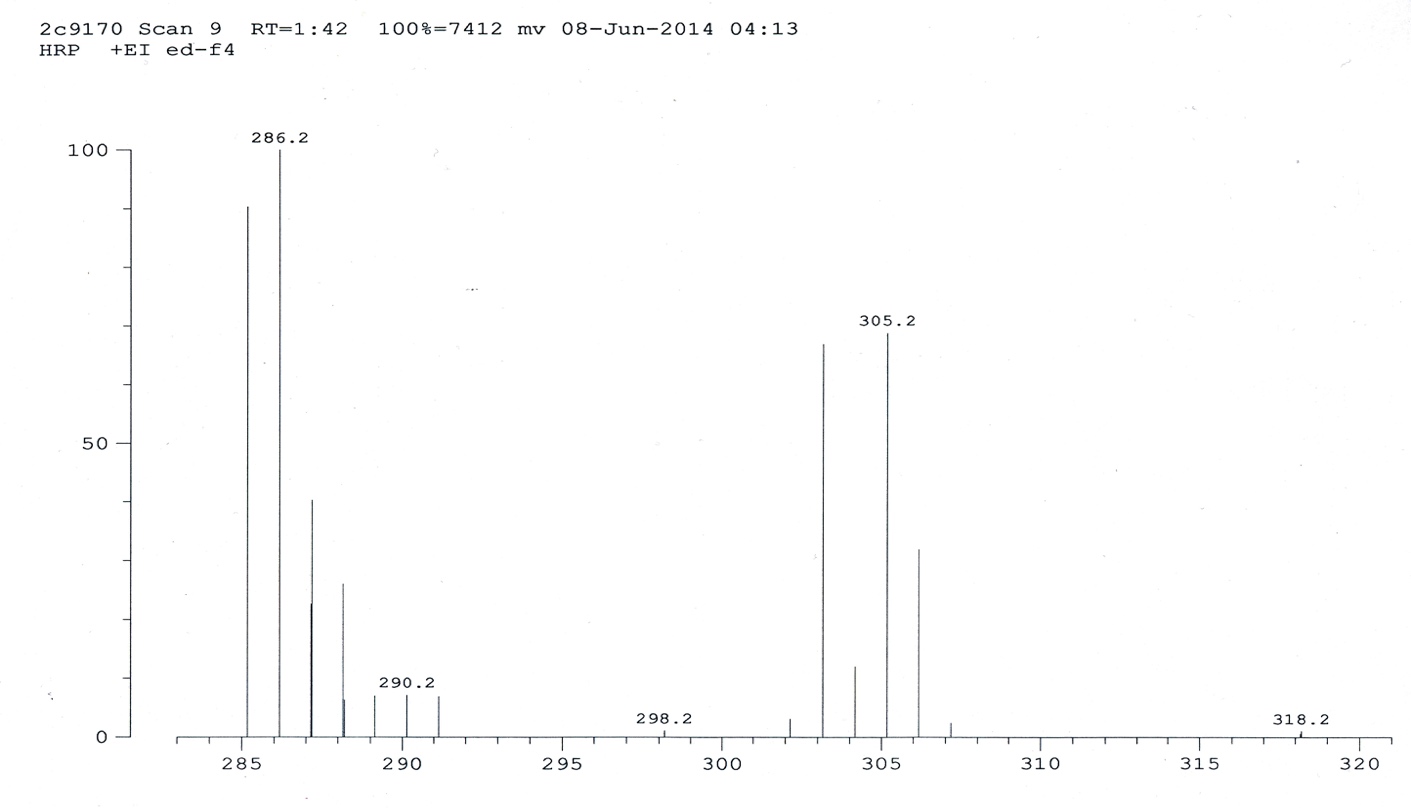
^**

**^1^H NMR spectrum of 2c in DMSO-d_6_**
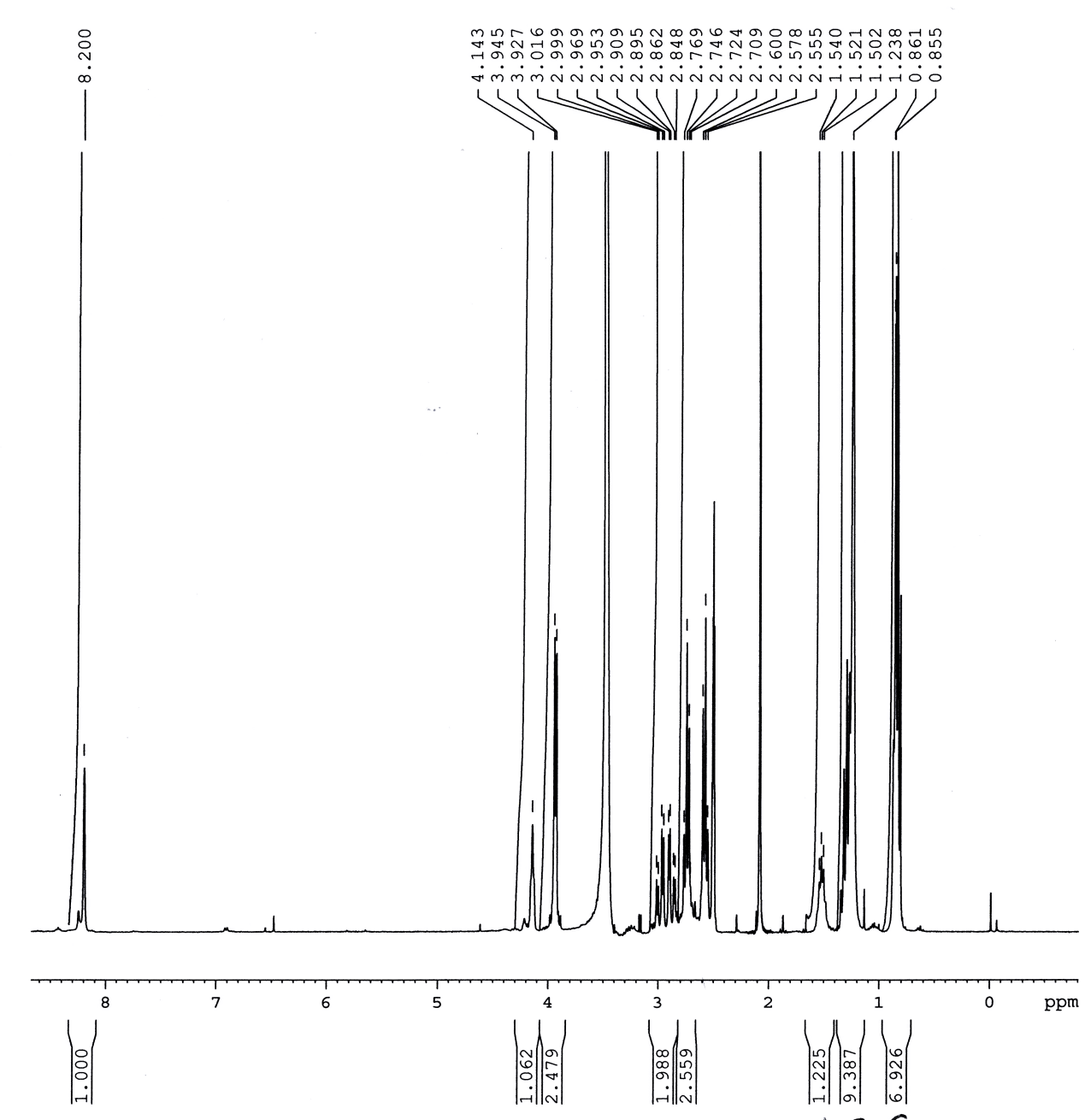


**^13^C NMR spectrum of 2c in DMSO-d_6_**


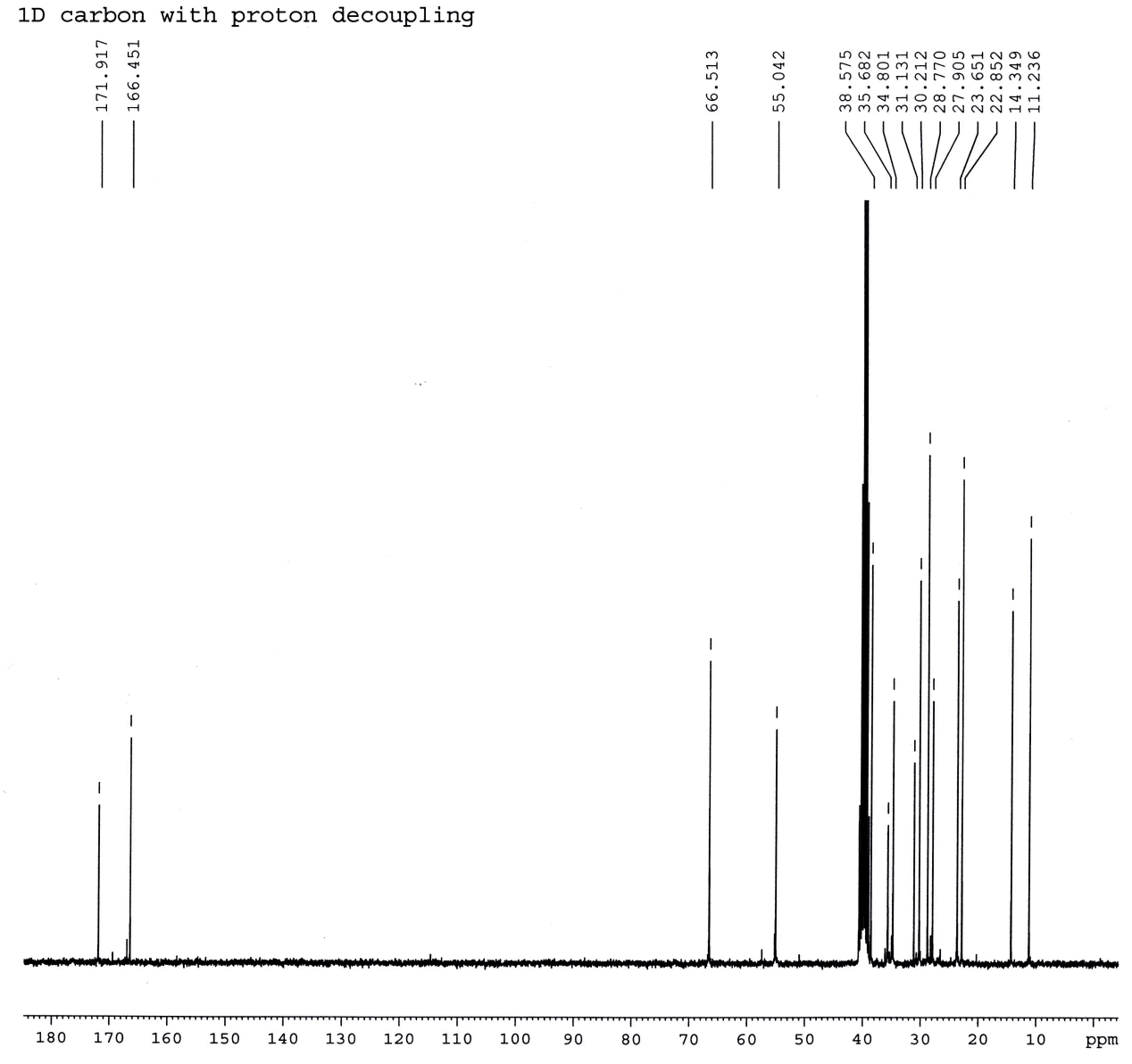


**MS of 2c**

# ^1^H NMR spectrum of 1d in DMSO-d_6_


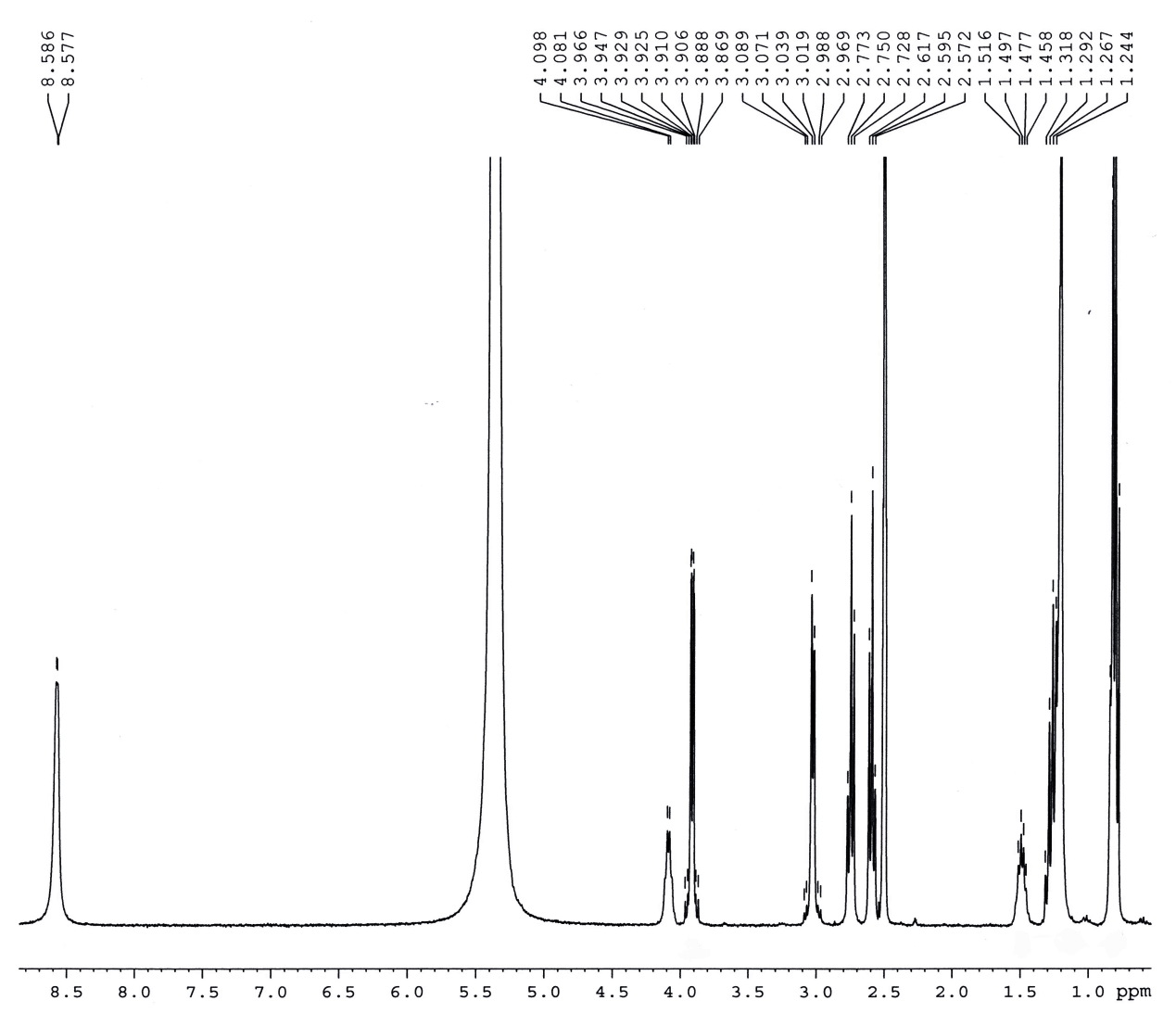


**^13^C NMR spectrum of 1d in DMSO-d_6_**

**
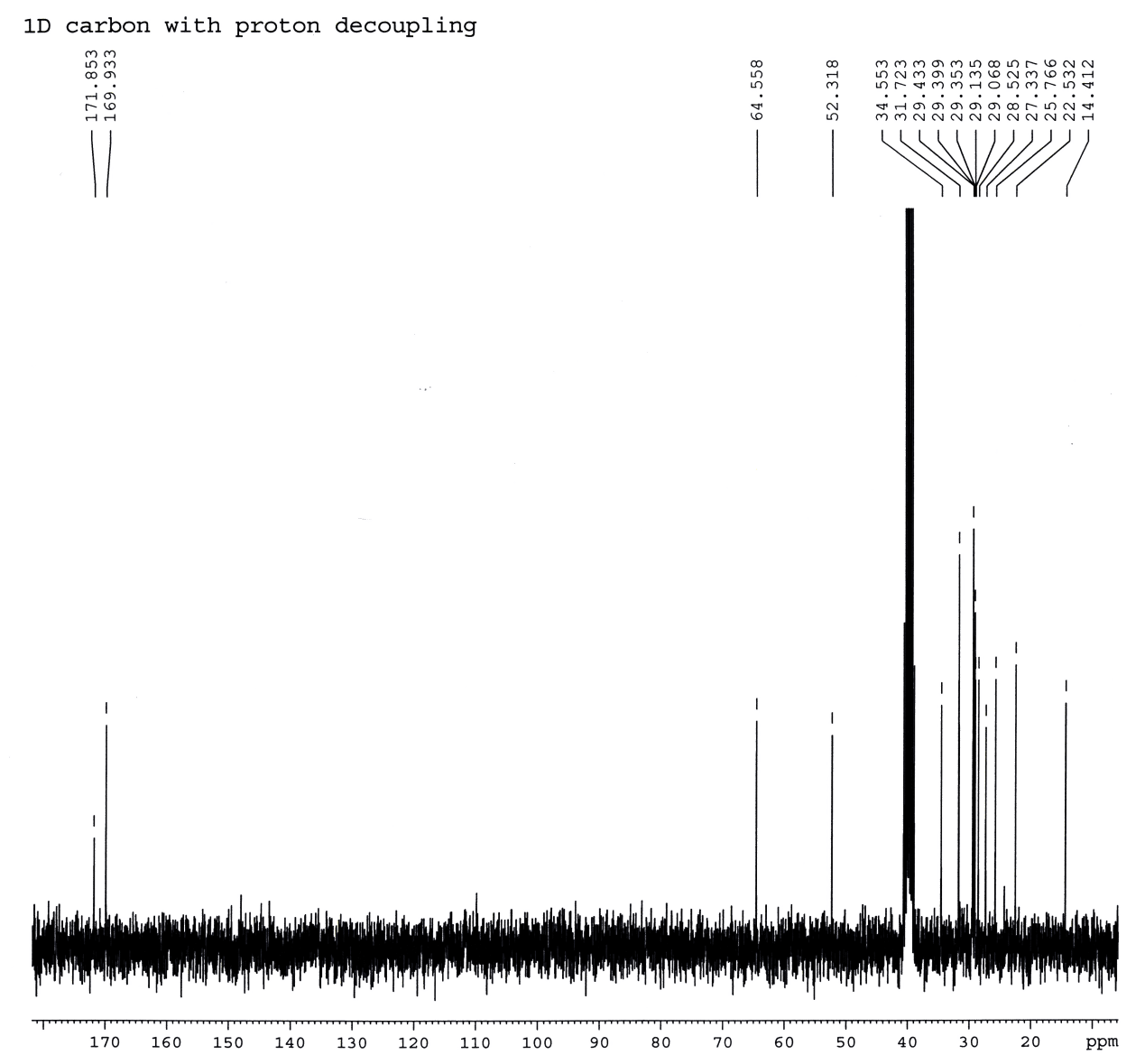
**

**MS of 1d**

**^1^H NMR spectrum of cis 2d in DMSO-d_6_**

**
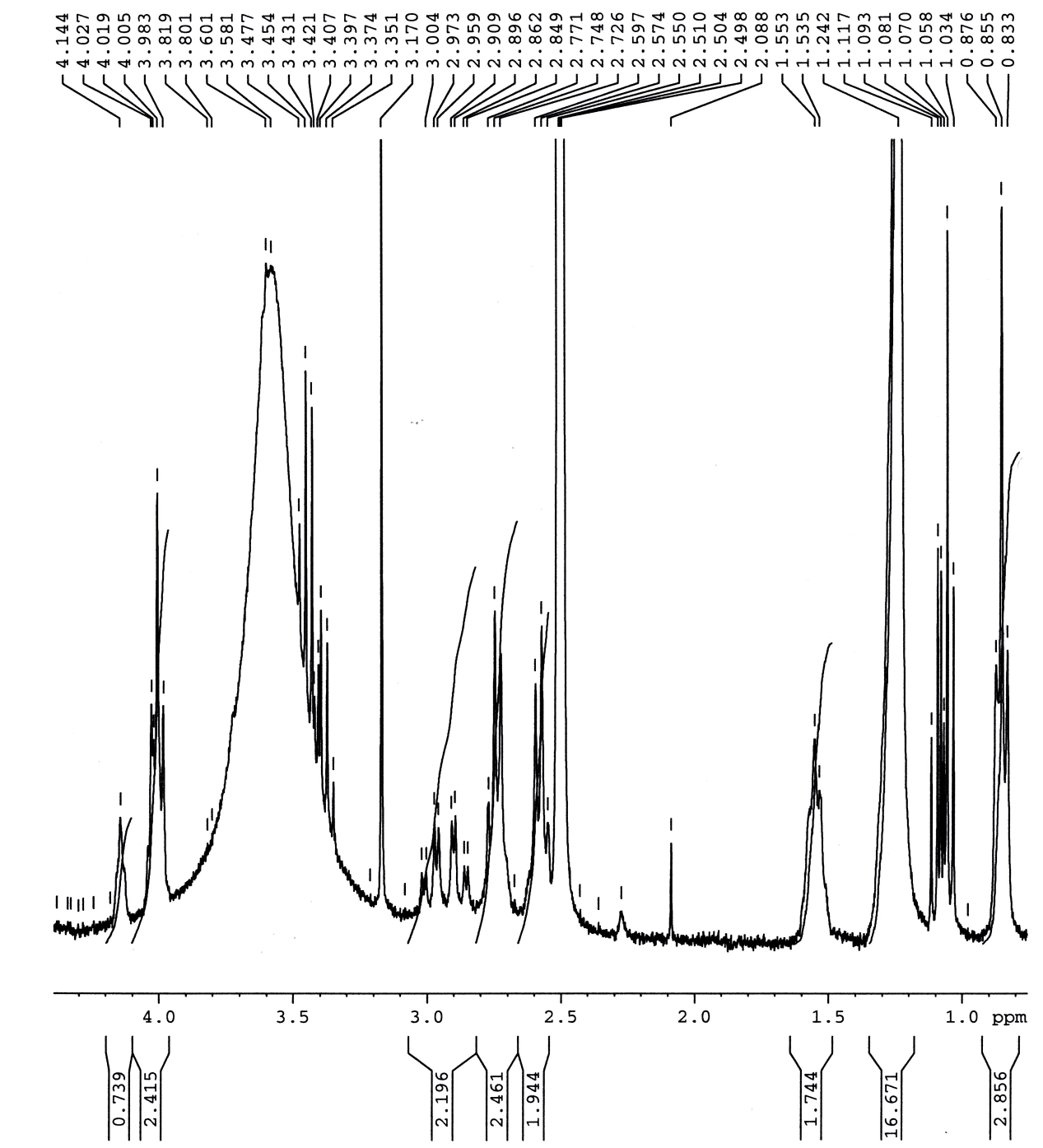
**

**^13^C NMR spectrum of cis 2d in DMSO-d_6_**

**
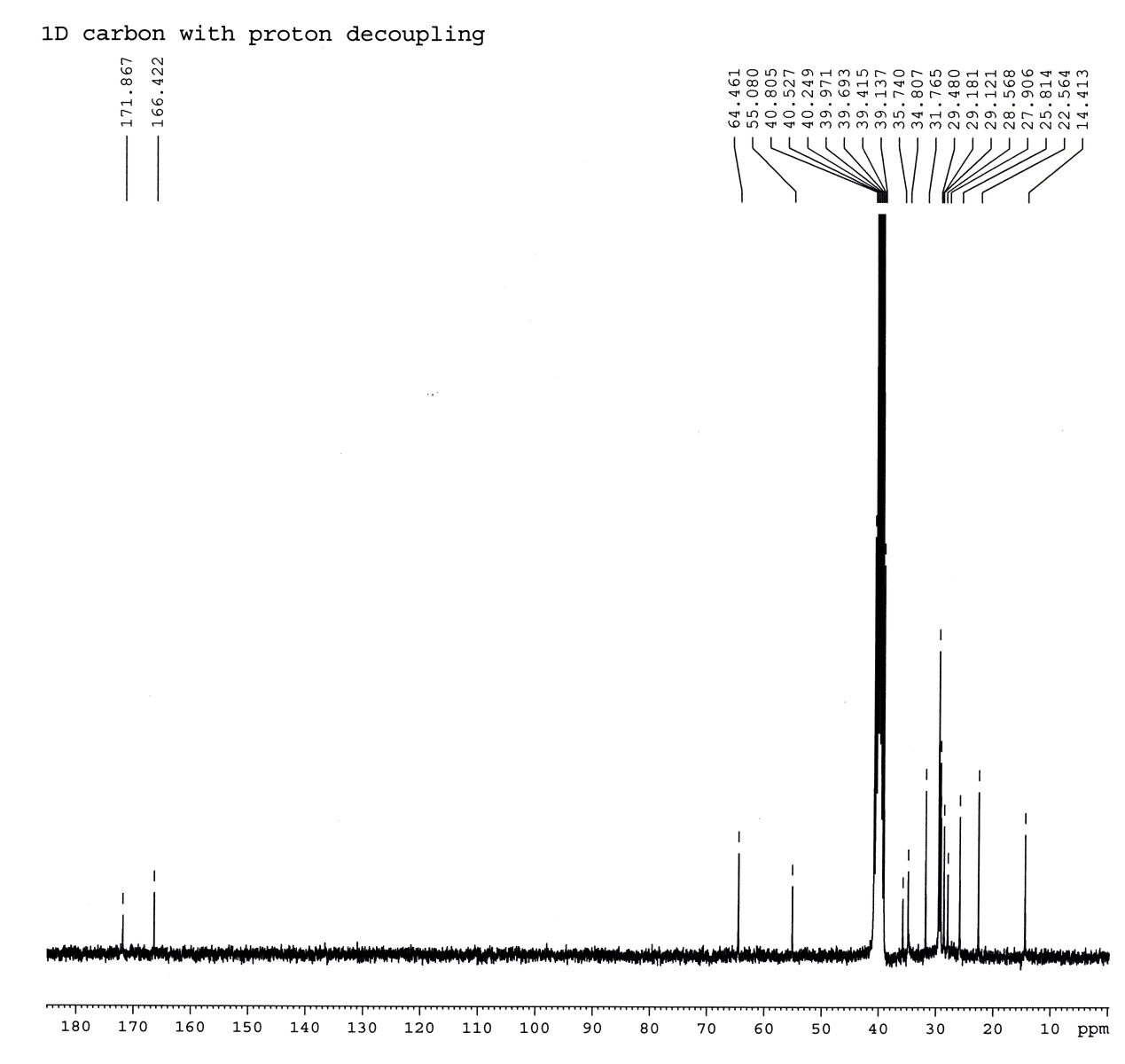
**

**^1^H NMR spectrum of 2d (cis:trans =3:1) in DMSO-d_6_**

**
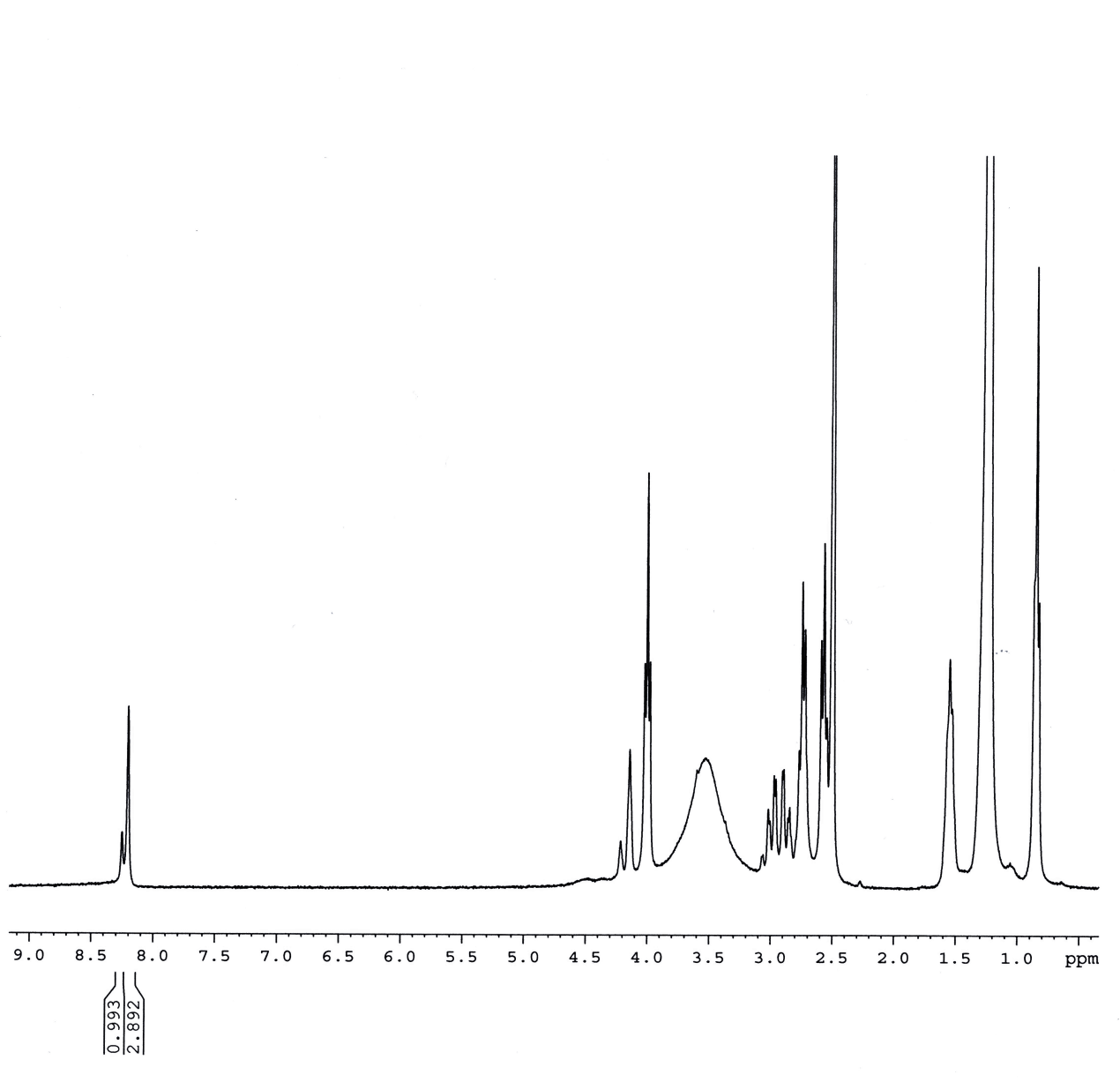
**

**^13^C NMR spectrum of 2d (cis:trans=3:1) in DMSO-d_6_**


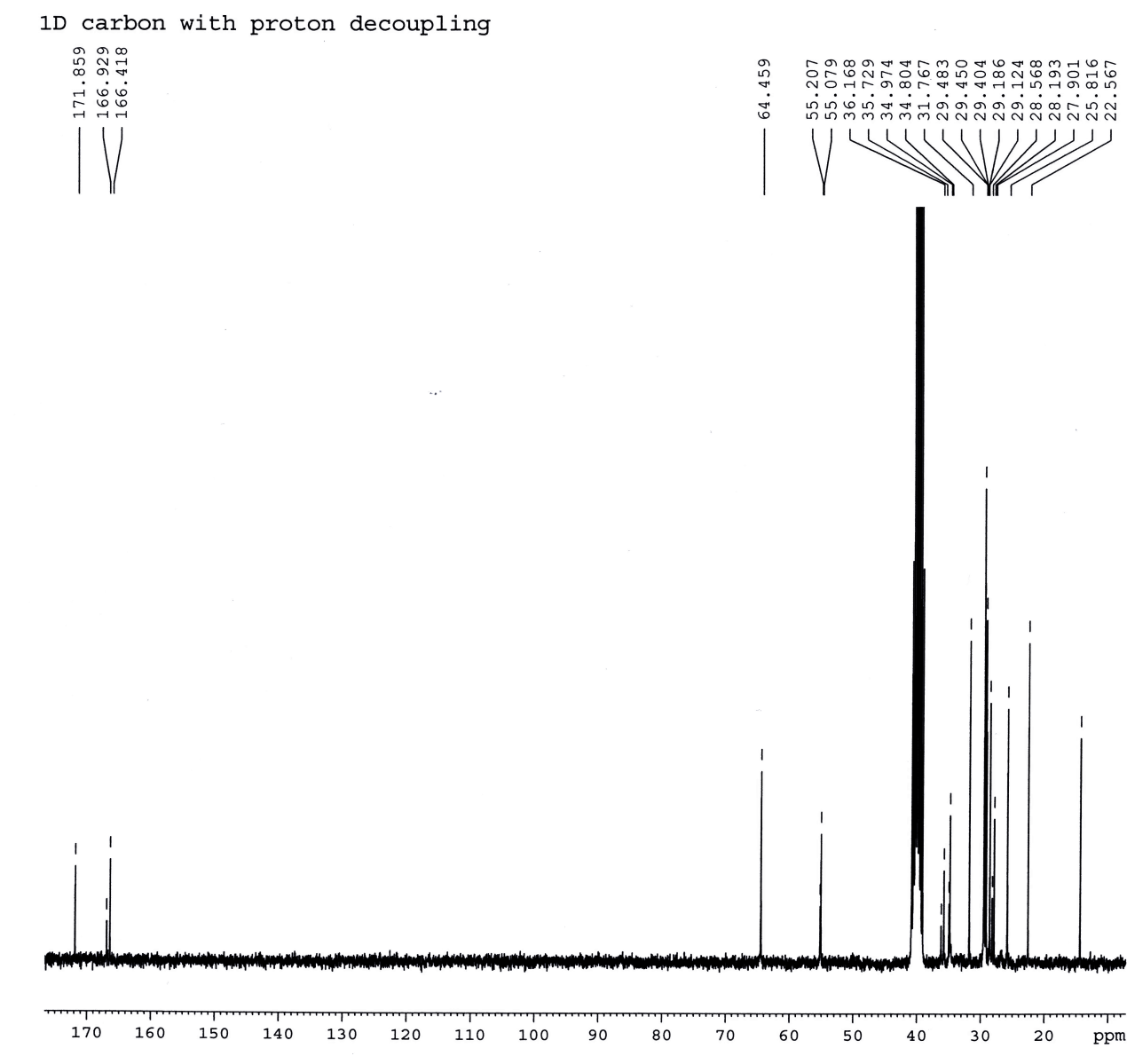


**MS of 2d**

# ^1^H NMR spectrum of 1e in D_2_O


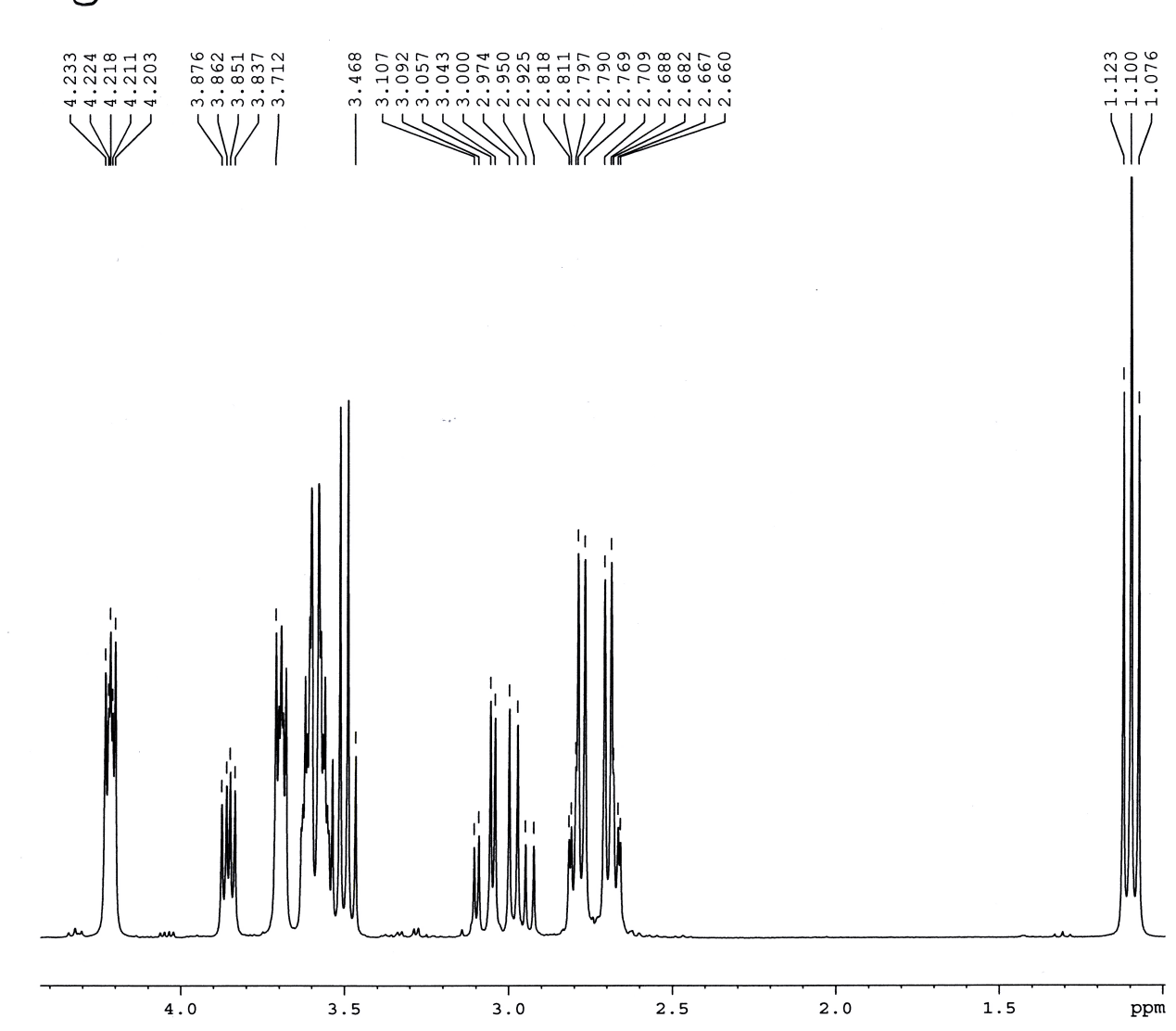


**^13^C NMR spectrum of 1e in D_2_O**


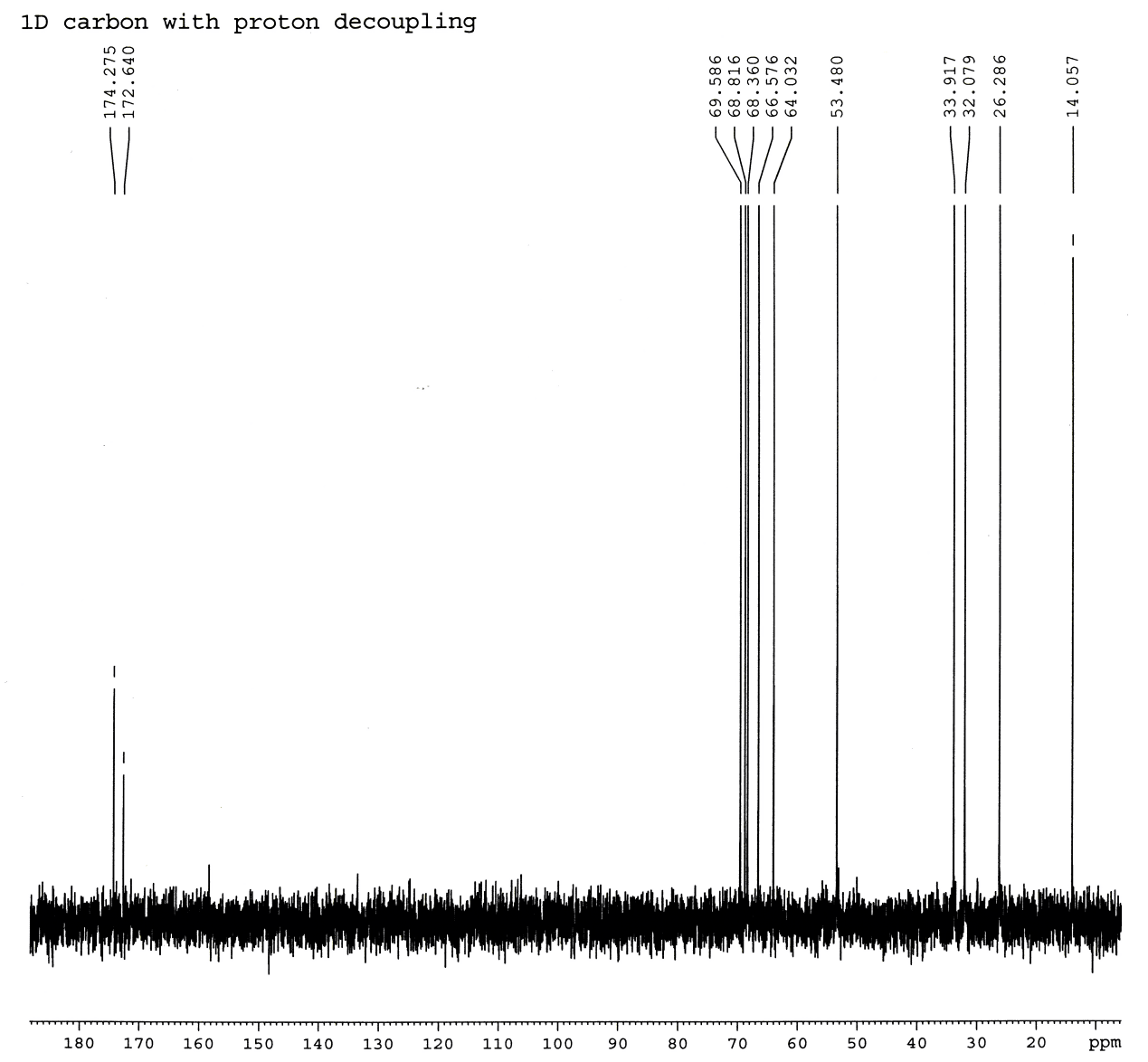


**MS of 1e**


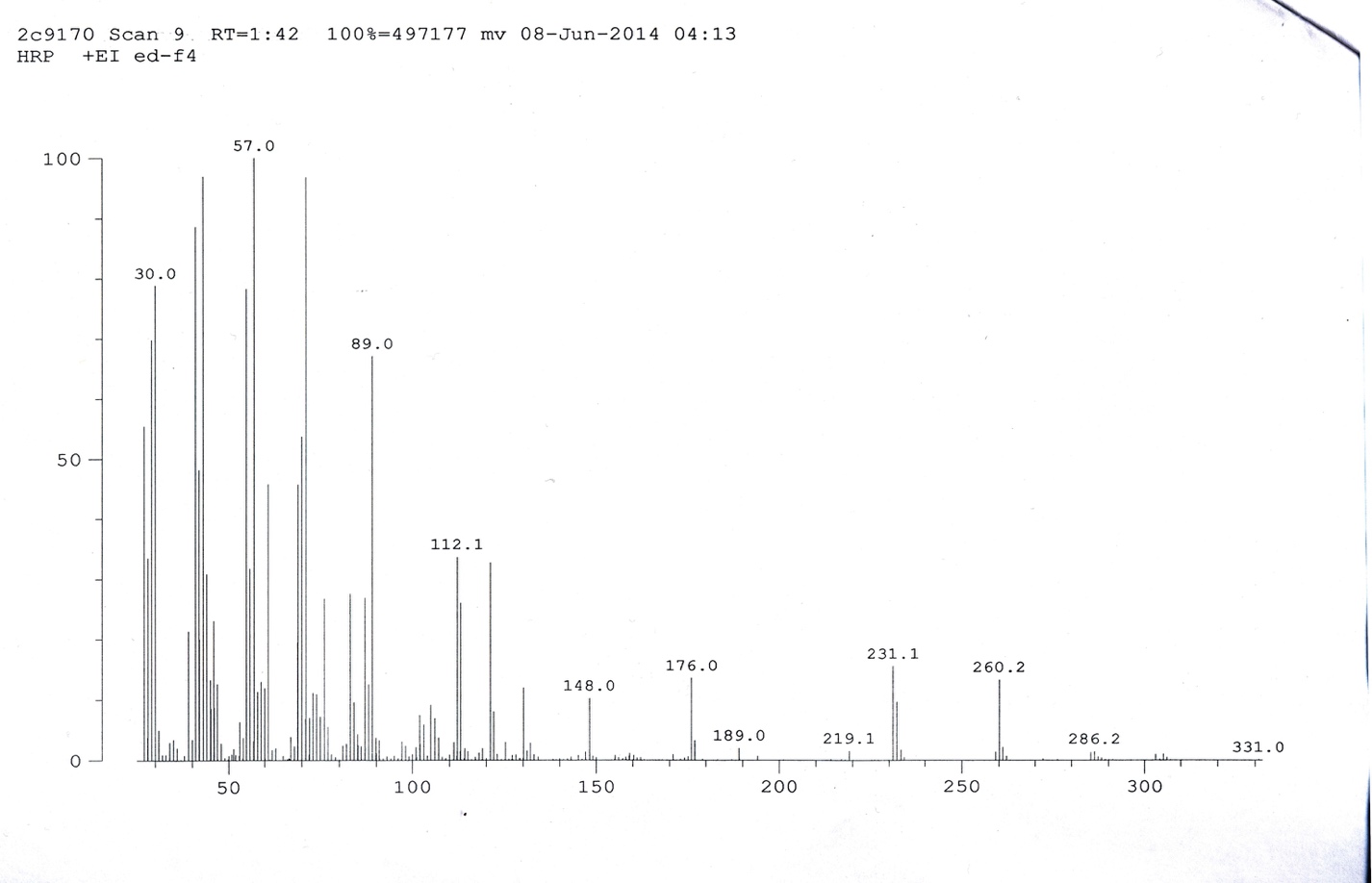


**^1^H NMR spectrum of cis 2e in DMSO-d_6_**


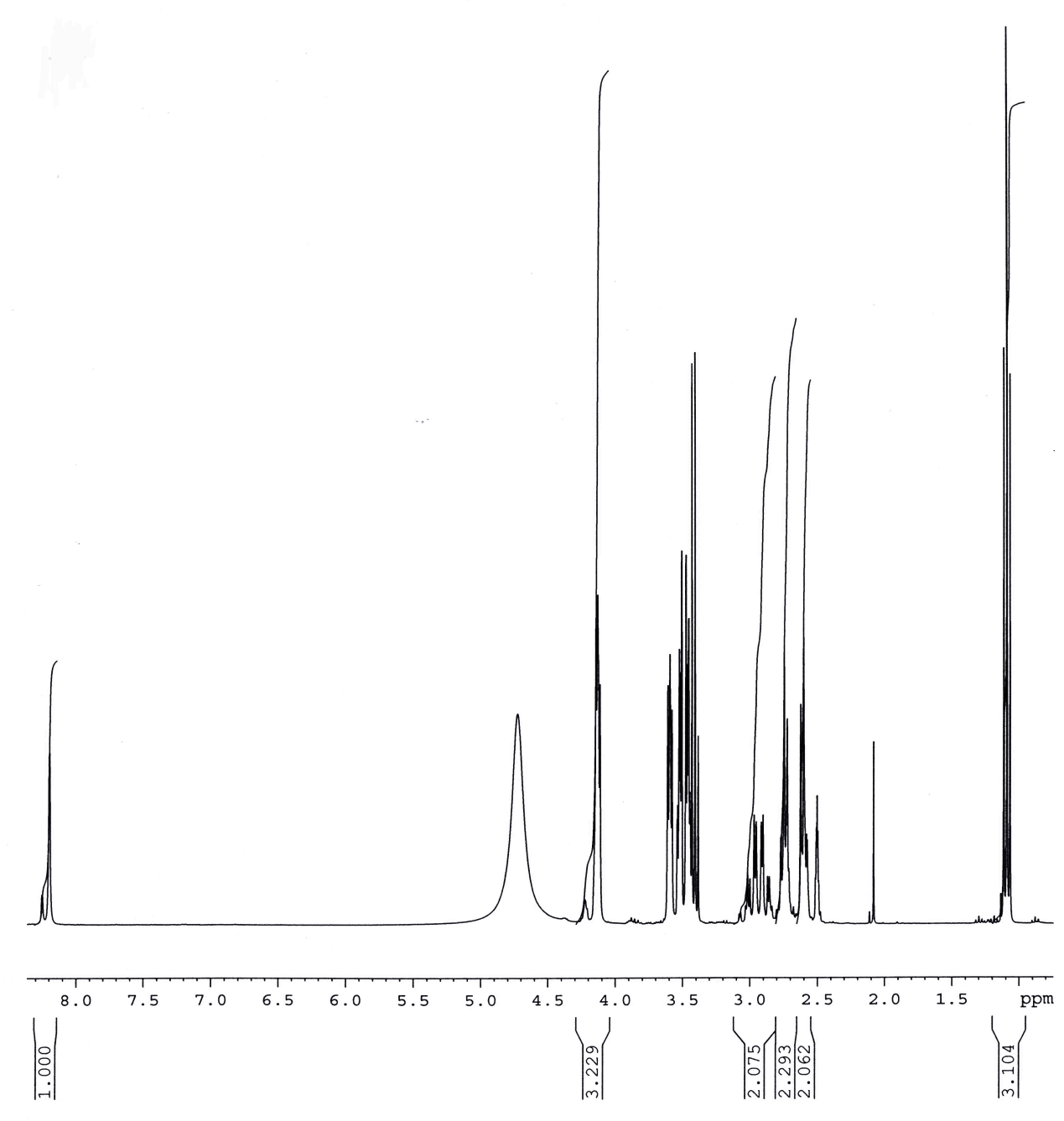


**^13^C NMR spectrum of cis 2e in DMSO-d_6_**


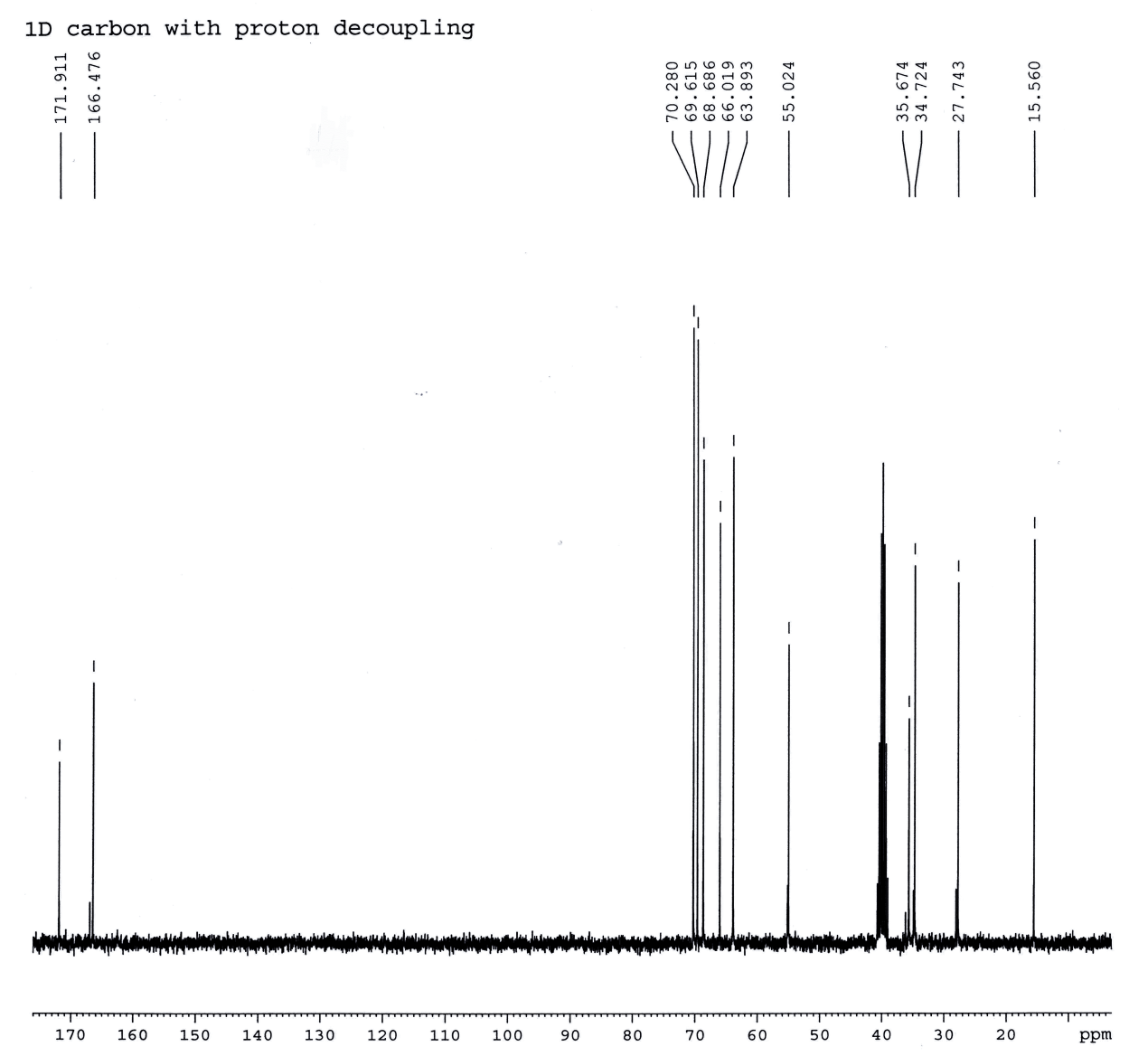


**^1^H NMR spectrum of trans 2e in DMSO-d_6_**


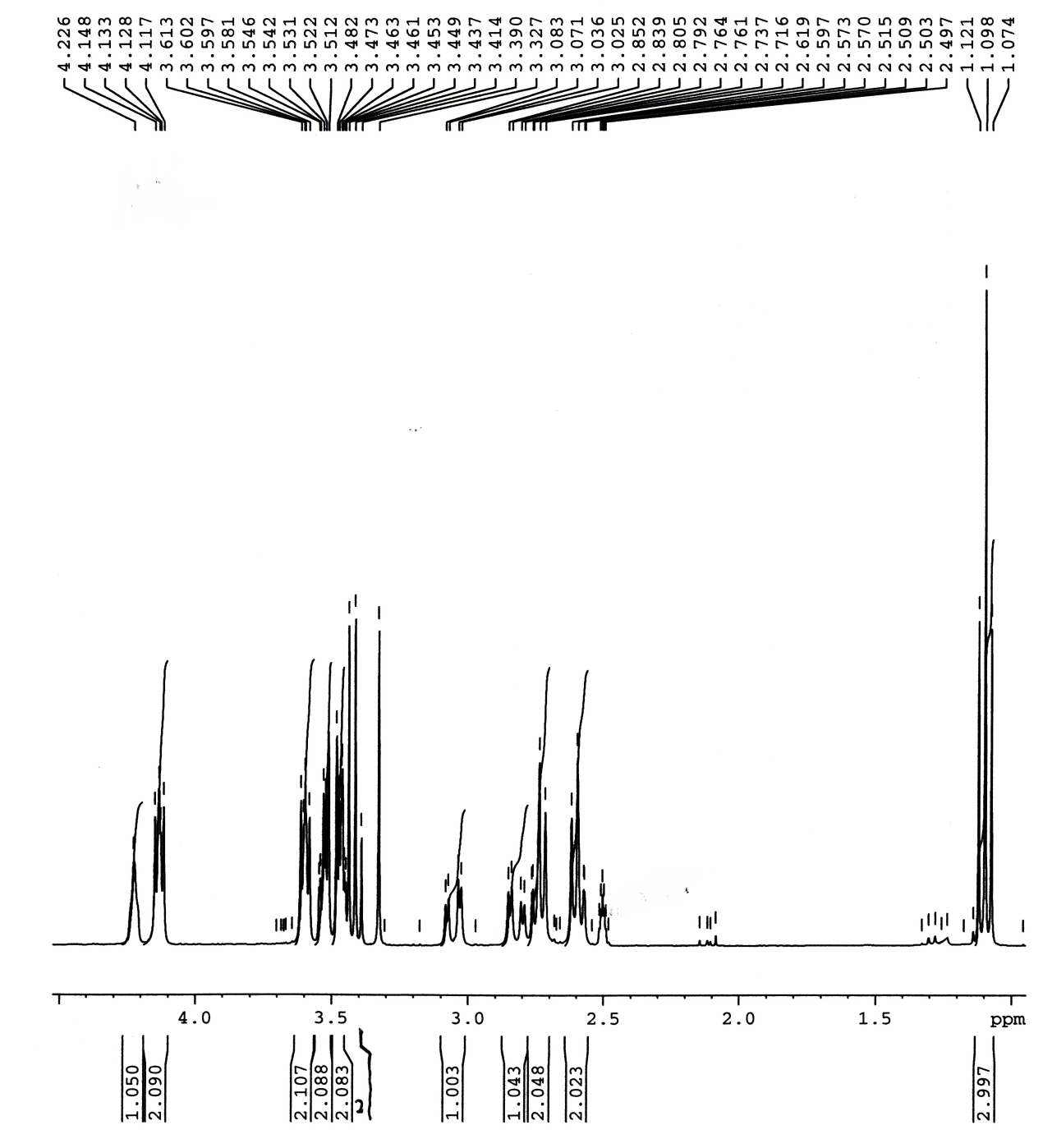


**^13^C NMR spectrum of trans 2e in DMSO-d_6_**


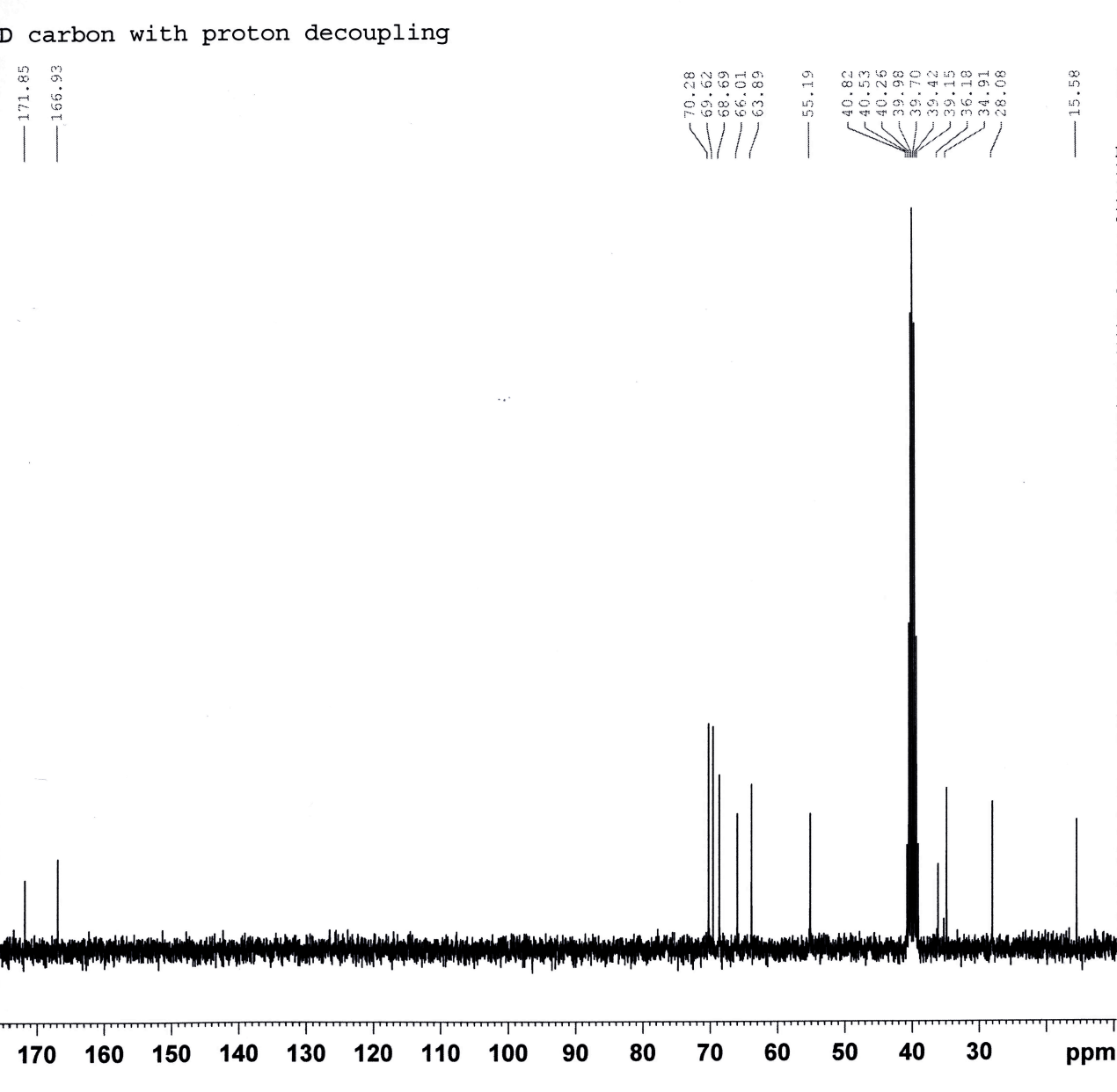

Supplement: Supporting Information [file rsos180272supp1.docx]
